# A Robust Pyrazolate Metal–Organic Framework for Efficient Catalysis of Dehydrogenative C–O Cross Coupling Reaction

**DOI:** 10.1021/jacs.4c03038

**Published:** 2024-05-09

**Authors:** Rong-Ran Liang, Zongsu Han, Peiyu Cai, Yihao Yang, Joshua Rushlow, Zhaoyi Liu, Kun-Yu Wang, Hong-Cai Zhou

**Affiliations:** Department of Chemistry, Texas A&M University, College Station, Texas 77843, United States

## Abstract

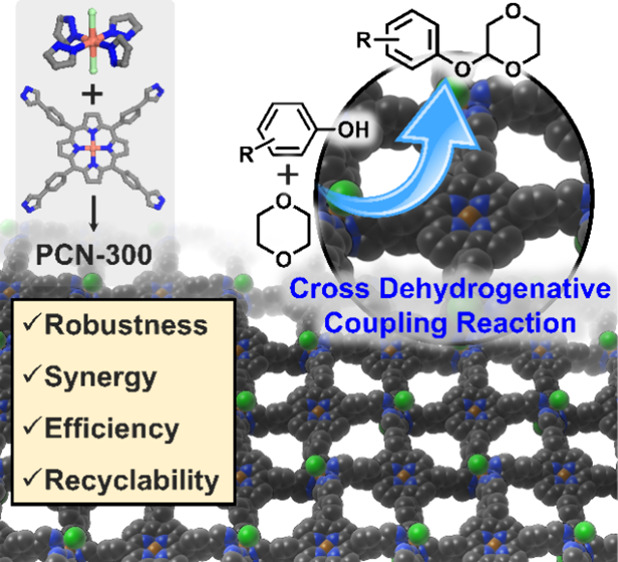

Construction of robust heterogeneous catalysts with atomic precision is a long-sought pursuit in the catalysis field due to its fundamental significance in taming chemical transformations. Herein, we present the synthesis of a single-crystalline pyrazolate metal–organic framework (MOF) named PCN-300, bearing a lamellar structure with two distinct Cu centers and one-dimensional (1D) open channels when stacked. PCN-300 exhibits exceptional stability in aqueous solutions across a broad pH range from 1 to 14. In contrast, its monomeric counterpart assembled through hydrogen bonding displays limited stability, emphasizing the role of Cu-pyrazolate coordination bonds in framework robustness. Remarkably, the synergy of the 1D open channels, excellent stability, and the active Cu-porphyrin sites endows PCN-300 with outstanding catalytic activity in the cross dehydrogenative coupling reaction to form the C–O bond without the “compulsory” *ortho*-position directing groups (yields up to 96%), outperforming homogeneous Cu-porphyrin catalysts. Moreover, PCN-300 exhibits superior recyclability and compatibility with various phenol substrates. Control experiments reveal the synergy between the Cu-porphyrin center and framework in PCN-300 and computations unveil the free radical pathway of the reaction. This study highlights the power of robust pyrazolate MOFs in directly activating C–H bonds and catalyzing challenging chemical transformations in an environmentally friendly manner.

## Introduction

Metal–organic frameworks (MOFs) are crystalline porous materials composed of organic linkers and inorganic metal ions/clusters, featuring high crystallinity, extensive porosity, tunable structures, and diverse functionalities.^1−3^ The versatility of MOFs has been demonstrated by their wide range of applications in fields such as gas storage/separation,^4,5^ catalysis,^6,7^ electronic devices,^8,9^ sensing,^9,10^ and drug delivery.^11,12^ Despite the significant progress made in MOF research heretofore, a pivotal challenge remains unresolved in numerous MOFs—the inherent framework vulnerability due to the mostly labile coordination bonds, which largely impedes their applications, especially under harsh conditions. Therefore, it is imperative to develop highly stable MOFs to meet the requirements arising from practical applications. The stability of an MOF hinges on a myriad of factors, yet it is predominantly believed that the dynamic coordination bonds bridging organic linkers and metal nodes determine the integrity of the framework under harsh conditions.

According to Pearson’s hard/soft acid/base (HSAB) principle, robust coordination bonds can be achieved through matching the softness/hardness of the Lewis acids/bases. For instance, probable robust combinations include carboxylate ligands with high-valent metal ions or borderline azolate ligands with borderline metal ions (Zn^2+^, Cu^2+^, Ni^2+^, Co^2+^, etc.).^13^ Compared to the carboxylate-based MOFs, which show stability in acidic and neutral solutions,^14,15^ azolate-based MOFs can even survive in strong alkaline solutions.^16−19^ In particular, the superior stability of pyrazolate-based MOFs (Pz-based MOFs), presumably originating from the highest p*K*_a_ (19.8) of pyrazole among those of all azoles,^20,21^ has aroused considerable interests recently. However, the high stability of Pz-based MOFs always corresponds to low crystallinity, presumably due to the inertness of the coordination bonds, thereby posing a significant challenge to an in-depth structural understanding via high-quality single-crystal diffraction studies. Thus far, several chemically stable Pz-based MOFs have been reported, such as Fe_2_(BDP)_3_, PCN-601, FDM-3, Co-BTTri, and BUT-32.^16,17,19,22,23^ Among these materials, different types of metal-containing secondary building units (SBUs) have been generated based on bidentate pyrazolate ligands including octahedral [M_4_(μ_4_-O)(μ-Pz)_6_], cubic [M_8_(μ_4_-OH)_6_(μ-Pz)_12_], triangular [M_3_(μ_3_-O)(μ-Pz)_3_(H_2_O)_3_]^+^, and double zigzag chain (M = Ni^2+^, Zn^2+^, and Co^2+^).^13^ However, using monocoordinated pyrazolates with mononuclear metal sites to construct two-dimensional (2D)-layered MOFs is largely unexplored,^24−26^ not to mention their stability, porosity, and potential as heterogeneous catalysts.

In the realm of organic synthesis, selective C–H activation to form C–O bonds holds paramount significance due to its wide application scope in synthesizing natural products and drug scaffolds.^27,28^ Yet, the direct cleavage of C–H bonds remains to be a challenging topic, as it often requires precious metal catalysts, prefunctionalization on substrates, and harsh synthetic conditions,^29−32^ raising concerns about cost and atom economy in the standpoint of green chemistry. In this context, the cross dehydrogenative coupling (CDC) reaction emerges as a powerful method that enables the direct activation of C–H bonds and the thermodynamically unfavorable removal of H_2_ molecules, thereby providing convenient, atomically economic, and environmentally friendly synthetic routes. While substantial progress has been made in C–C bond formation via CDC reactions,^33−35^ studies on constructing C–O bonds are still rare.^36,37^ Recent studies have explored the etherification of sp^3^ C–H bonds in cyclic ethers and phenols using homogeneous or heterogeneous Cu-based catalysts. Nevertheless, these reactions typically necessitate *ortho*-position directing groups on the phenols, limiting their substrate scope and versatility.^38−40^ Thus, efforts to discover new heterogeneous catalysts for CDC reactions with high selectivity, long cycle lives, and broad applicability are highly desired.

Compared to other heterogeneous catalyst supports like porous silica^41,42^ and porous polymers,^43,44^ MOFs stand out as ideal candidates for catalysis due to their facile installation of active sites and well-defined crystal structures.^45,46^ In this work, we present the design and synthesis of an ultrastable Cu-Pz-based MOF, denoted as PCN-300 (PCN = Porous Coordination Network), which is constructed from the judiciously chosen porphyrinic ligand 5,10,15,20-tetrakis(4-(1*H*-pyrazol-4-yl)-phenyl)porphyrin (H_4_TPPP). Particularly, Cu incorporated into the porphyrin center and formed the octahedral [CuPz_4_Cl_2_] SBU simultaneously, resulting in a lamellar structure with two distinct copper sites and one-dimensional (1D) open channels when stacked. Remarkably, PCN-300 displays exceptional stability in aqueous solutions with pH values ranging from 1 to 14. In contrast, its monomeric counterpart (complex-TPPP), a porphyrinic network assembled through noncovalent interactions with Cu anchoring into the porphyrin center exclusively, exhibits limited stability, highlighting the contribution of Cu-Pz coordination bonds to the overall framework robustness. Most notably, PCN-300 showcases outstanding catalytic activity in the CDC reaction of *para*-substituted phenols and *p*-dioxane with a yield up to 96%, demonstrating a superior performance coupled with remarkable recyclability and sustainability compared to homogeneous Cu-porphyrin catalysts. Control experiments reveal the synergy between the Cu-porphyrin center and framework in PCN-300 and computations unveil a free radical pathway of the reaction. This study not only exemplifies the power of stable Pz-based MOFs in catalyzing challenging organic transformations but also demonstrates the synergistic effects between the MOF framework and active sites for efficient catalysis.

## Results and Discussion

### Synthesis and Structure Analysis of PCN-300 and Complex-TPPP

**Figure 1:**
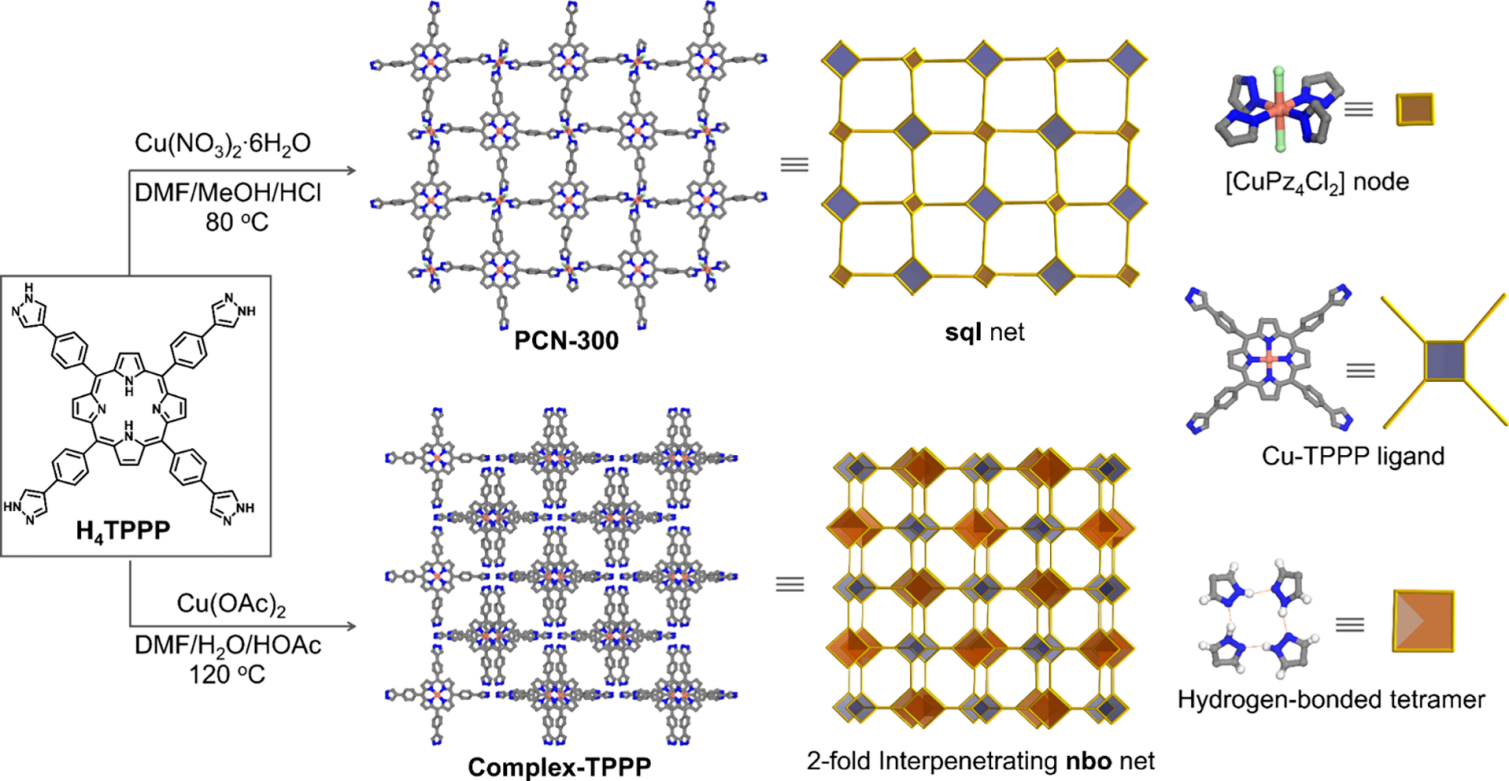
Schematic illustration of synthesizing PCN-300 and complex-TPPP under different conditions and their corresponding single-crystalline structures and topologies. C, N, Cl, and Cu atoms are represented by gray, blue, green, and orange, respectively. Hydrogen atoms in the structures are removed for clarity.

**Figure 2:**
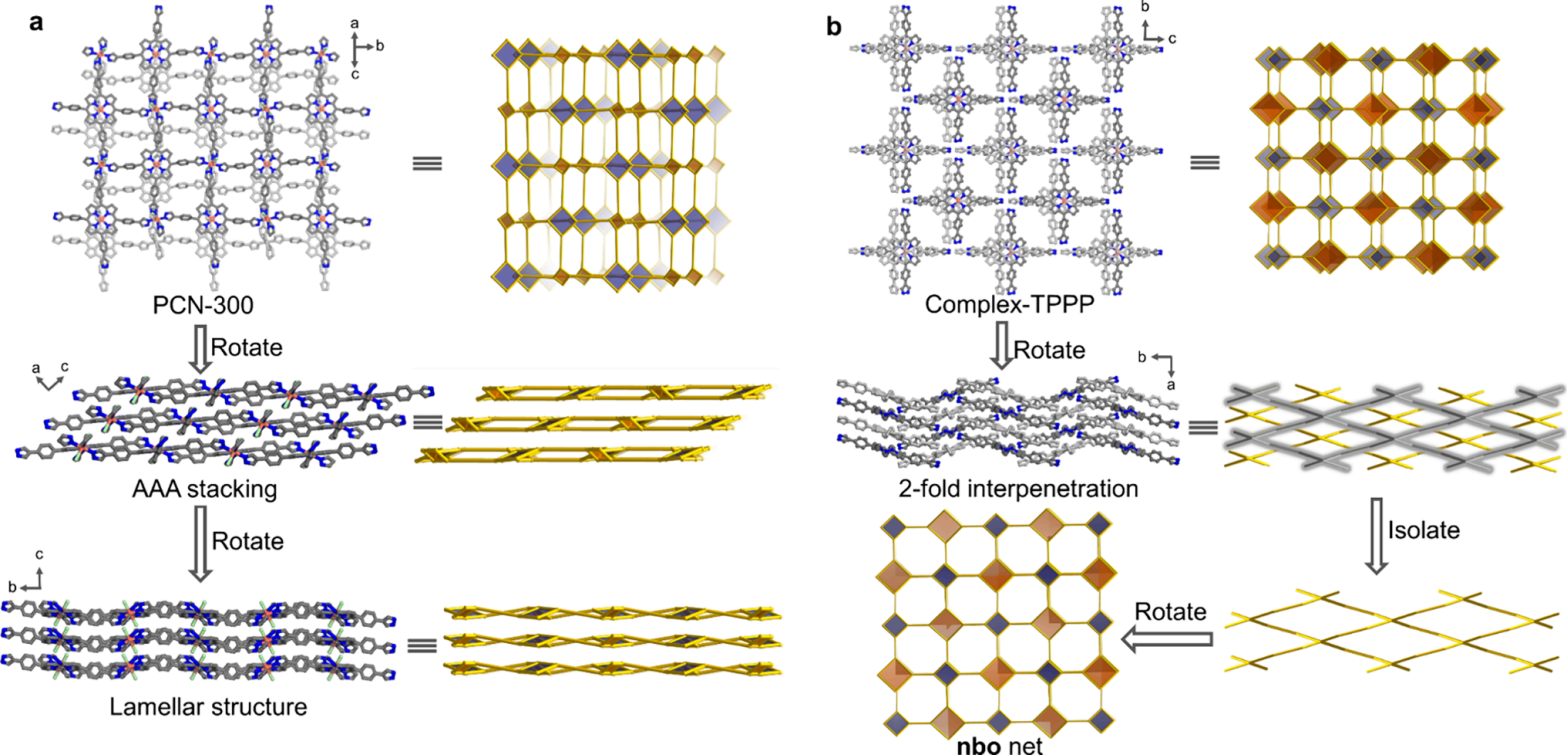
Illustration of the packing structures and topologies of (a) PCN-300 and (b) complex-TPPP. C, N, Cl, and Cu atoms are represented by gray, blue, green, and orange, respectively. Hydrogen atoms in the structures are removed for clarity.

PCN-300 was synthesized through a solvothermal reaction involving Cu(NO_3_)_2_·6H_2_O and H_4_TPPP in a mixture of *N*,*N*-dimethylformamide (DMF), methanol (MeOH), and hydrochloric acid (HCl) (Figure 1). A single-crystal X-ray diffraction (SCXRD) study revealed its crystalline structure in the *C*2/*m* space group. The lattice parameters were determined as *a* = 18.9931(18) Å, *b* = 26.081(3) Å, *c* = 6.9278(7) Å, and α = γ = 90°, β = 104.377(7)° (Table S1). The structural analysis elucidated the presence of an independent (4,4)-c **sql** network within the Cu(Cu-TPPP)Cl_2_ formulation, forming a lamellar aggregate (Figure 2a). Within this framework, Cu^2+^ ions were found to anchor at two distinct positions: the center of the porphyrin and the metal node. Each metal node, depicted as [CuPz_4_Cl_2_], exhibited an octahedral coordination geometry bonded with four nitrogen atoms originating from distinct ligands and two chlorine atoms. Furthermore, the ligand can serve as a bridge to connect four Cu nodes to form an extended 2D framework (Figure 1). These 2D layers further stack in an inclined AAA manner through π···π and C–H···π interactions, separated at an interlayer distance of 6.9 Å (Figure 2a and Figure S1). Consequently, such a stacking arrangement resulted in a porous framework with 1D open channels of nearly square shape (∼12 Å) aligned parallel to the *c*-axis (Figure S2). Notably, the void space within the synthesized PCN-300 was filled with disordered solvent molecules, occupying approximately 30% of the unit cell volume (Figure S3). The large void coupled with the well-defined 1D channels enhances the mass transfer, making PCN-300 promising for applications in heterogeneous catalysis.

To study the role that the Cu-Pz linkage played in the integral properties and applications of the resulting material, a monomeric counterpart of PCN-300, termed complex-TPPP, was synthesized through a solvothermal reaction involving Cu(OAc)_2_ and H_4_TPPP in a mixture of DMF, water, and acetic acid (HOAc) (Figure 1). SCXRD study revealed that complex-TPPP was crystallized in a *Pnna* space group, with *a* = 8.1112(3) Å, *b* = 26.5911(8) Å, and *c* = 27.8600(10) Å (Table S1). Interestingly, Cu^2+^ ions were found to be exclusively anchored into the center of the porphyrin, while the four pyrazole groups from four distinct ligands assembled through multiple hydrogen bonds instead of coordination bonds with Cu (Figure S4). In contrast to PCN-300, the four ligands are interconnected in a nonplanar manner (Figure S5), leading to the formation of a 2-fold interpenetrated **nbo** network (Figure 2b). Complex-TPPP displays 1D open channels (∼11 Å) along the *a*-axis, which occupy approximately 35% of the unit cell volume (Figure S6).

### Porosity and Stability Analysis

**Figure 3:**
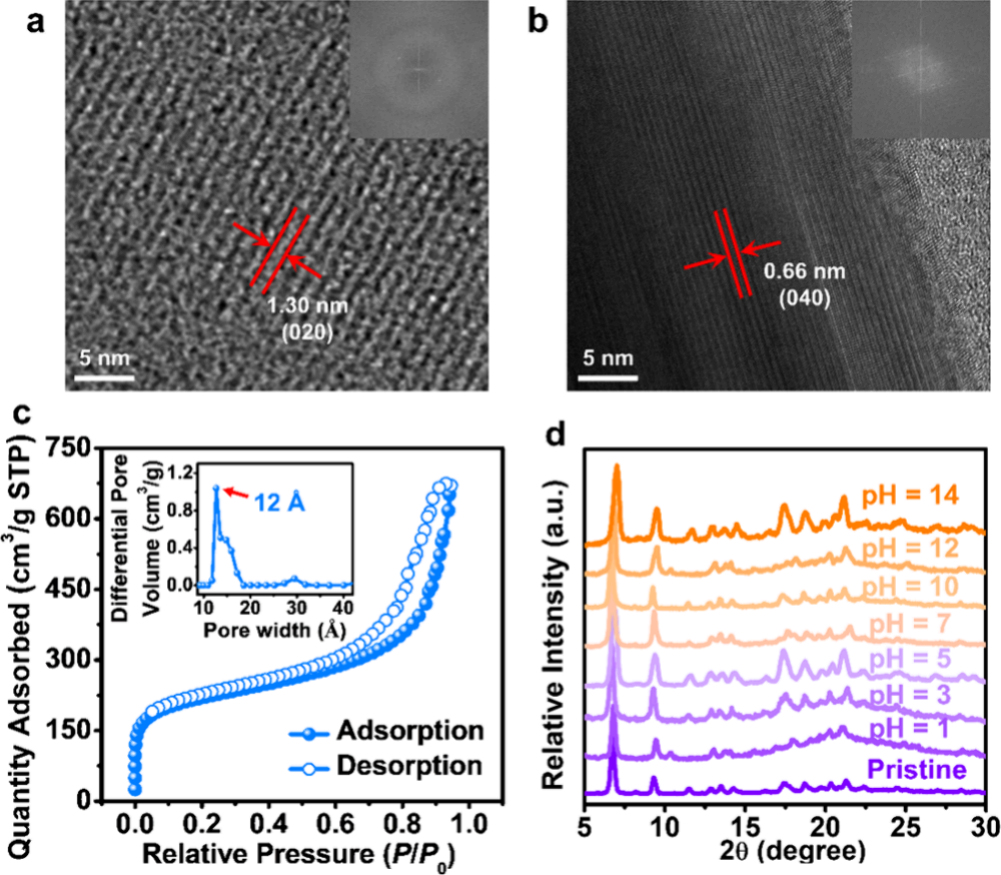
High-resolution TEM images of (a) PCN-300 and (b) complex-TPPP. The insets show the fast Fourier transformation (FFT) patterns. (c) Nitrogen sorption isotherm of PCN-300. The inset shows the pore size distribution profile. (d) PXRD patterns of PCN-300 before and after immersion in aqueous solutions with pH values ranging from 1 to 14.

Powder samples were produced from large-scale synthesis. The high crystallinity of the powder samples of both PCN-300 and complex-TPPP were verified by the high-resolution transmission electron microscopy (TEM) images (Figure 3a,b and Figure S7), which revealed their highly ordered structures. X-Ray photoelectron spectroscopy (XPS) measurements were further conducted for PCN-300 and complex-TPPP to investigate their elemental composition. The XPS survey spectra revealed the presence of Cu, N, Cl, and C in PCN-300 (Figure S8), wherein complex-TPPP exhibited elements Cu, N, and C (Figure S9). Moreover, the high-resolution scans over the Cu 2p spectral region of both PCN-300 and complex-TPPP identified the peaks corresponding to Cu 2p_3/2_ and Cu 2p_1/2_, appearing at binding energies near 934.0 and 954.0 eV, respectively. The shakeup peaks at 943.5 and 962.5 eV were indicative of the presence of Cu(II) in both PCN-300 and complex-TPPP.

The porosity of PCN-300 was evaluated through nitrogen sorption measurement conducted at 77 K. The isotherm displayed sharp adsorption in the low-pressure range (Figure 3c), indicating the presence of micropores within the material. The Brunauer–Emmett–Teller (BET) and Langmuir surface areas of PCN-300 were calculated from the adsorption isotherm to be 788 and 940 m^2^g^–1^ (Figure S10), respectively. Additionally, the total pore volume, assessed at *P*/*P*_0_ = 0.95, was measured as 1.04 cm^3^g^–1^, providing compelling evidence of the permanent porosity of PCN-300. The pore size distribution calculated by the DFT method indicated a pore size of ∼1.2 nm (Figure 3c), matching well with the pore dimension measured from its single-crystal data. The permanent porosity of PCN-300 is anticipated to significantly facilitate the mass transfer in catalytic reactions. However, a limited nitrogen uptake of 54 cm^3^g^–1^ at 0.95 *P*/*P*_0_ was observed for complex-TPPP, and its BET and Langmuir surface areas were calculated to be 62 and 75 m^2^g^–1^, respectively (Figure S11). The observed low surface area can be attributed to the partial pore collapse after guest solvent removal originating from the lability of the hydrogen bonds within complex-TPPP.

Thermogravimetric analysis (TGA) was conducted to assess the thermal stability of PCN-300. The resulting curve disclosed a decomposition temperature of ∼300 °C (Figure S12), suggesting its high thermal stability. To validate the chemical stability of PCN-300, it was immersed in a series of aqueous solutions with pH values ranging from 1 to 14. According to the powder X-ray diffraction (PXRD) results, the crystallinity of PCN-300 can be partially maintained even under the harsh conditions of pH = 1 or 14 (Figure 3d), demonstrating its robustness. Besides, slight shifts in some PXRD peaks were observed, which could be attributed to sliding between the 2D MOF layers and distortions within the layers. In contrast, complex-TPPP showed limited stability with the appearance of broad peaks in its PXRD pattern upon desolvation (Figure S13), which can be attributed to the lability of the hydrogen bonds, providing further evidence for its limited N_2_ adsorption. These results highlight the crucial role of Cu-Pz coordination bonds in the construction of robust frameworks.

### Catalysis Study

**Figure 4:**
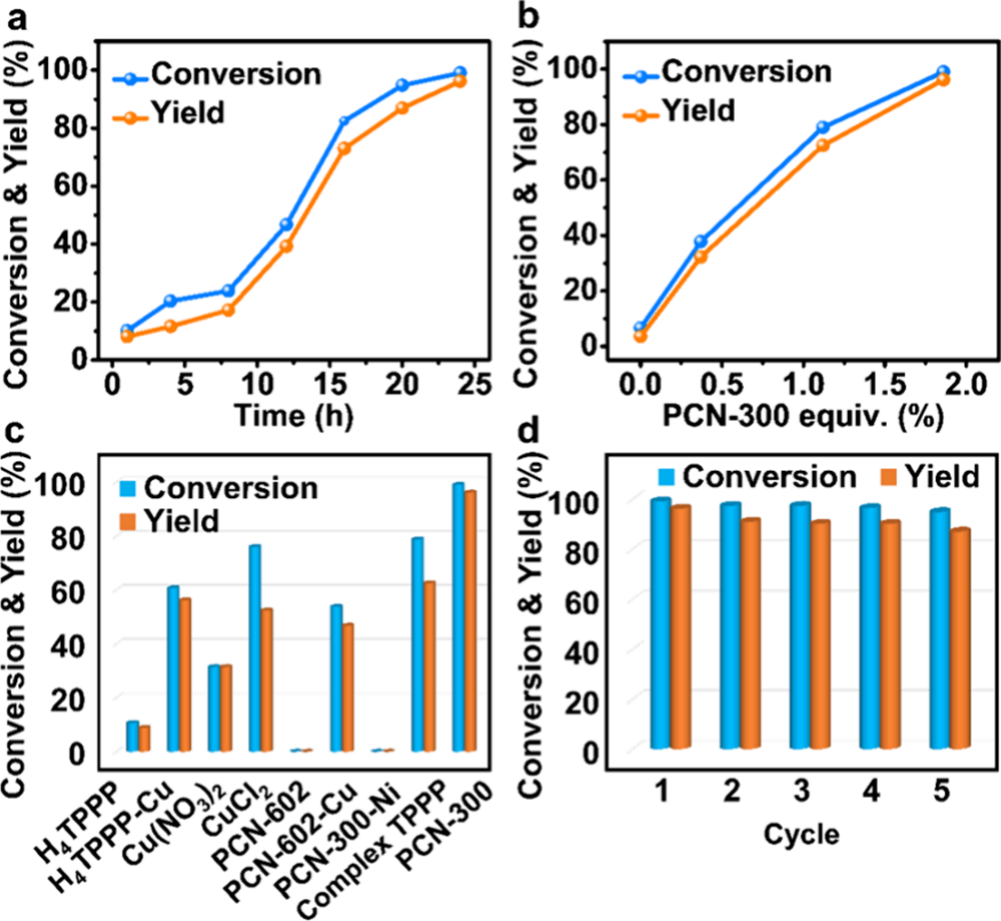
(a) Reaction kinetics of the PCN-300-catalyzed CDC reaction. (b) The reaction conversion and yield versus the percentage of the PCN-300 catalyst. (c) The catalytic performance of different catalysts. (d) The cycling performance of PCN-300 as a heterogeneous catalyst. Note: The reactions were conducted under the optimal reaction condition of **1** (0.25 mmol), **a** (2 mL), PCN-300 (1.86 mol %), and DTBP (5.66 equiv) for 24 h at 120 °C, except for the control variables.

In light of the presence of the active Cu sites coupled with its 1D open channels and high chemical stability, PCN-300 was employed as a heterogeneous catalyst in the CDC reaction, and interestingly, it enabled the formation of C–O bonds on phenol substrates without *ortho*-position directing groups. Initial investigations focused on assessing the catalytic activity of PCN-300, with methyl 4-hydroxybenzoate (**1**) and *p*-dioxane (**a**) chosen as substrates in a model reaction. The preliminary catalytic experiment was performed in the presence of di-*tert*-butyl peroxide (DTBP, 5.66 equiv) with PCN-300 (1.86 mol %) as the catalyst at 120 °C. Remarkably, PCN-300 delivered the desired cross coupling product, methyl 4-(1,4-dioxan-2-yloxy)benzoate (**1a**), reaching a yield of 87% after 20 h (Figure 4a). Encouraged by this promising result, we further optimized the reaction conditions to improve the yield. In particular, a systematic study was conducted on regulating the dosage of the PCN-300 catalyst, the equivalent of DTBP, and the reaction time (Figure 4a,b and Figures S14–S17), resulting in high yield up to 96%. Notably, PCN-300 not only exhibits superior performance in terms of yield and selectivity when compared with various homogeneous Cu-porphyrin catalysts^47,48^ but also demonstrates high conversion and yield parallel with the reported heterogeneous catalysts for CDC reactions with *ortho*-position directing groups on the phenols (Table S2).^39,40,49^

To further understand the catalytic activity exhibited by PCN-300, we conducted a series of control experiments using different catalysts under the optimal condition of **1** (0.25 mmol), **a** (2 mL), catalyst (1.86 mol %), and DTBP (5.66 equiv) for 24 h at 120 °C. Initially, H_4_TPPP, H_4_TPPP-Cu, Cu(NO_3_)_2_·6H_2_O, and CuCl_2_ were directly employed as catalysts for the CDC reaction (Figure S18). While the three Cu-based catalysts demonstrated catalytic activity in the CDC reaction, their yields are comparatively lower than that of PCN-300. The catalytic yield of the porphyrin compound H_4_TPPP is negligible (Figure 4c), suggesting the pivotal role of Cu in the reaction. This conclusion was further validated by the experimental results when applying PCN-602,^18^ consisting of Ni-porphyrin ligand and Ni-Pz node, as the catalyst, which showed no desired product. Furthermore, when replacing the Ni-porphyrin in PCN-602 with Cu-porphyrin (PCN-602-Cu) (Figure S19), significant enhancement was observed in the catalytic activity, and the yield increased from 0 to 47%.

An additional facet of interest lies in evaluating the impact of the other Cu center, the [CuPz_4_Cl_2_] node, on the catalytic performance of PCN-300. Herein, a counterpart of PCN-300, PCN-300-Ni, was synthesized through the combination of H_4_TPPP-Ni and Cu(NO_3_)_2_·6H_2_O (Figure S20), which showed high thermal stability and permanent porosity as revealed by its TGA analysis and nitrogen sorption measurement (Figures S21 and S22). As an isostructural MOF of PCN-300, PCN-300-Ni consists of Ni-porphyrin centers and [CuPz_4_Cl_2_] nodes. XPS measurement was conducted to investigate its elemental composition, which indicates the presence of Cu, Ni, N, Cl, and C in PCN-300-Ni (Figure S23). Surprisingly, no desired product was detected when PCN-300-Ni was used as the catalyst (Figure 4c), indicating that the Cu-porphyrin center is the key to catalyze the CDC reaction, while the [CuPz_4_Cl_2_] node displays no catalytic activity. This finding, coupled with the significant enhancement in catalytic activity of PCN-300 when compared to the complex H_4_TPPP-Cu and another MOF PCN-602-Cu, suggests that the framework of PCN-300 can provide a synergistic effect, thereby facilitating the reaction and elucidating its impressive catalytic performance.

In addition, given its structural similarity to PCN-300, the catalytic activity of complex-TPPP was also assessed in the CDC reaction. It showed a moderated yield (63%) due to the presence of Cu-porphyrin sites, yet a lower efficiency compared to PCN-300 attributed to the lack of the synergistic effect from the framework and limited porosity for mass transfer. Moreover, to gain a deeper insight of the role of Cu centers in PCN-300 on the CDC reaction, we further performed the hot filtration test by filtering the model reaction mixture of methyl 4-hydroxybenzoate (**1**) and *p*-dioxane (**a**) through a preheated Celite pad after the reaction for 4 h, during which a 20% conversion was achieved. Subsequently, the filtrate was further treated and monitored under the same conditions for an additional 12 h (Figure S24). Remarkably, no further conversion was detected (Figure S25), indicating that substrate turnover ceases after filtration. This result suggests the absence of leaching of catalytically active Cu sites, thereby confirming the mechanism of heterogeneous catalysis and highlighting the high stability of PCN-300.

Furthermore, to test the recyclability of PCN-300 in the coupling reactions, the catalyst was centrifuged, isolated at the end of each reaction, and subsequently reused for successive runs. Remarkably, the catalytic activity remained intact for at least five cycles without a discernible decrease in yield, demonstrating the excellent recyclability of this MOF catalyst (Figure 4d and Figure S26). It should be noted that complex-TPPP failed to be recycled due to its limited stability. Following the optimal reaction condition, we extended the substrate scope of the MOF catalyst. The PCN-300-catalyzed CDC reactions proceeded well with *para*-monosubstituted phenols containing −COOMe, −CN, or −CHO moieties and *p*-dioxane (Table 1 and Figures S27–S29). Remarkably, these substrates can lead to desired products in yields ranging from moderate to excellent (52–96%). Although the result obtained from a phenol containing aldehyde group was not ideal, one possible reason is that the aldehyde group was not compatible with the oxidation condition. Consistent with previous studies, halogen-substituted phenols failed to yield detectable products (Figure S30). Exploration of bisubstituted phenols with −COOMe and −CF_3_ moieties revealed good yields (89–94%) (Figures S31 and S32). In summary, the established reaction condition exhibited compatibility with phenols bearing −COOMe, −CN, and −CF_3_ groups, as well as multisubstituted phenols.

**Table 1:**
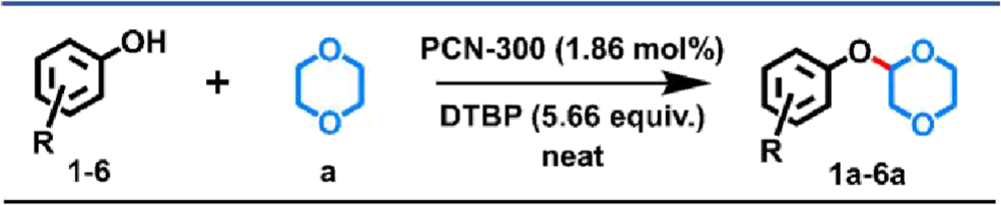

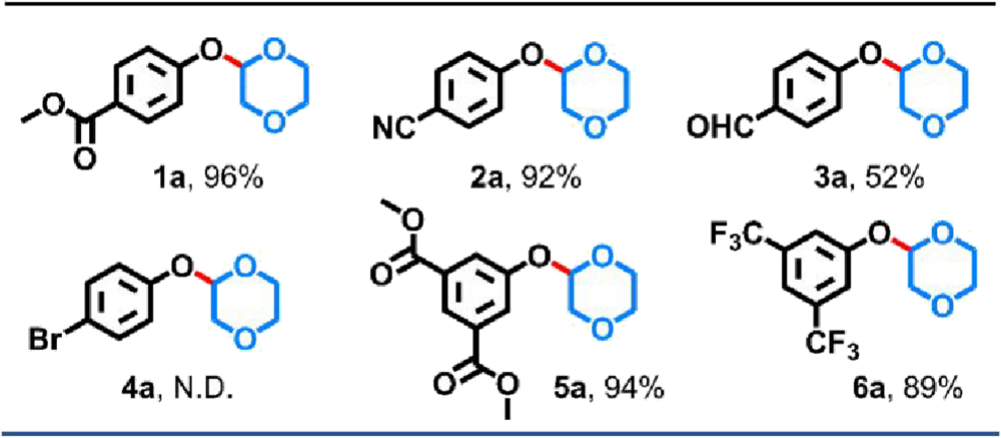
Substrate Scope of Phenols in PCN-300-Catalyzed CDC Reactions^a^

a Reaction condition: **1**–**6** (0.25 mmol), **a** (2 mL), PCN-300 (1.86 mol %), DTBP (5.66 equiv) for 24 h at 120 °C isolated yield based on phenol. N.D. = not detected.

### Mechanism Study

**Figure 5:**
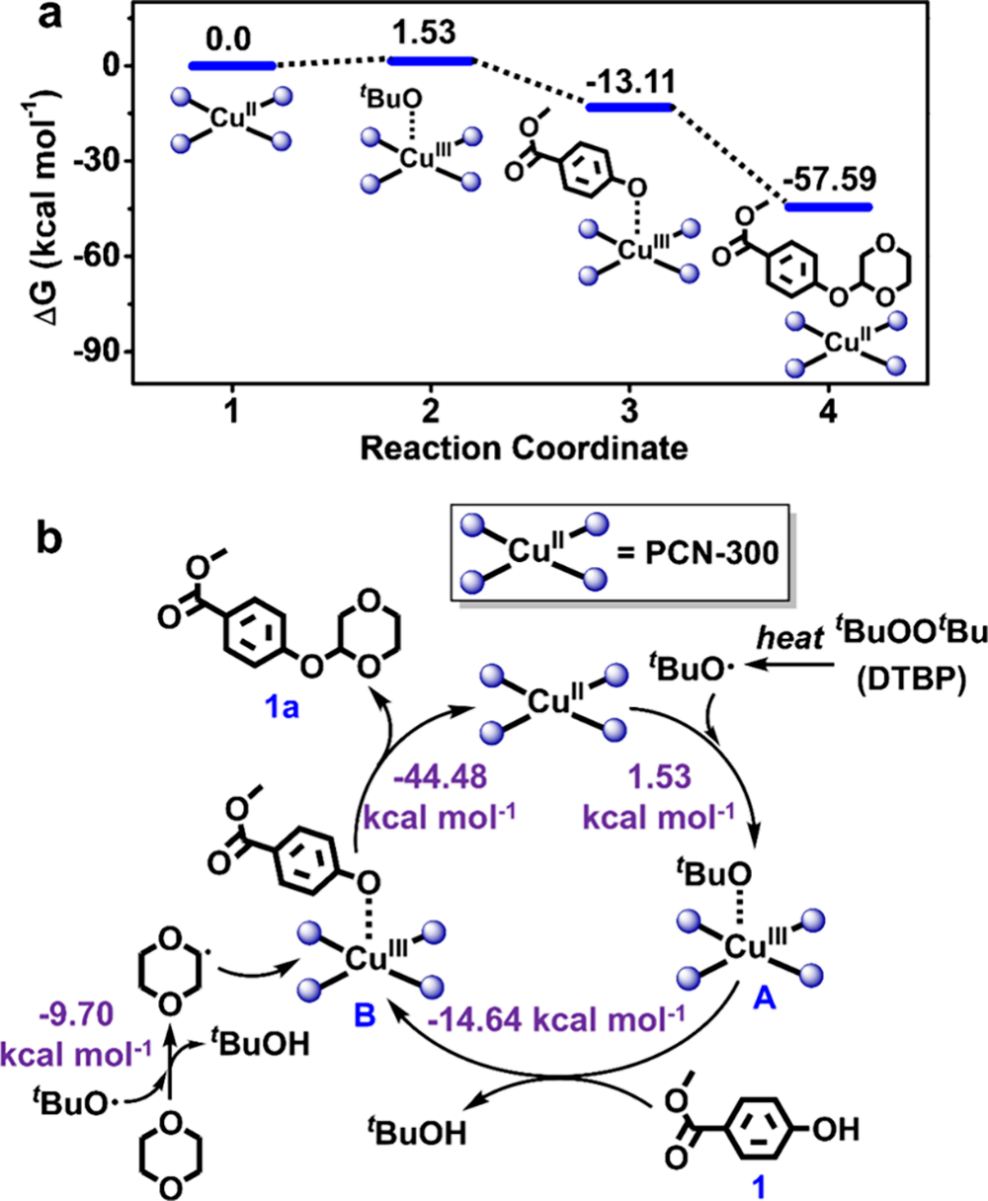
(a) Gibbs free energy profile of the CDC reaction. The free energy of each intermediate state is relative to the free energy (zero) of the Cu-porphyrin in PCN-300. (b) Proposed reaction pathway for the CDC reaction with PCN-300 as the heterogeneous catalyst.

Scanning electron microscopy (SEM) measurement was performed on the samples of PCN-300 and complex-TPPP before and after catalysis. The morphology of PCN-300 appeared irregular, with some particles exhibiting a rhombic geometry, and it remained almost identical after the catalytic cycles. Similarly, the sphere morphology of complex-TPPP was consistent before and after catalysis (Figure S33). These results suggest that the catalytic process has a negligible effect on the morphology of these heterogeneous catalysts. To deepen the understanding of the reaction mechanism, a control experiment was conducted by adding the free radical scavenger, butylated hydroxytoluene (BHT), to the reaction system. As a result, the desired product was not detected in the presence of BHT (Figure S34), indicating a free-radical-involved pathway of the reaction. This finding coincided with the mechanism reported for the homogeneous reaction.^47,48^ DFT calculations using the Cu-porphyrin unit as the catalytic model are further performed to figure out the free-radical-involved mechanism. As shown in Figure 5a and Figure S35, two possible intermediate states are proposed, and the corresponding Gibbs free energies of the intermediates and product are calculated, which revealed the CDC reaction to be an energetically favorable process.

Based on the experimental results and theoretical calculations, a plausible reaction pathway in the heterogeneous reaction is proposed (Figure 5b). Initially, the peroxide bond in DTBP undergoes homolysis at the reaction temperature to produce a *tert*-butoxyl free radical (^*t*^BuO·) and consequently generates ^*t*^BuO-Cu(III) complex A, which is endergonic with a Gibbs free energy change of +1.53 kcal mol^–1^. Intermediate A further reacts with compound **1** to produce intermediate B with a Gibbs free energy change of −14.64 kcal mol^–1^, suggesting that this step is exergonic. The reaction of the *p*-dioxane free radical, initiated by ^*t*^BuO· (Δ*G* = −9.70 kcal mol^–1^), with intermediate B releases desired product **1a** and the PCN-300 catalyst to finish the catalytic cycle. The final step is also exergonic by −44.48 kcal mol^–1^. As a result, the CDC reaction to form a C–O bond over PCN-300 is energetically favorable, in which the formation of intermediate A is a limiting step with the largest uphill Gibbs energy change.

## Conclusions

In conclusion, we demonstrate the synthesis of an ultrastable Cu-Pz-based MOF, PCN-300, bearing two distinct types of copper sites: the Cu-porphyrin center and octahedral [CuPz_4_Cl_2_] SBU, which exhibits a lamellar structure with 1D-stacked open channels. Different from its monomeric counterpart assembled through hydrogen bonding, PCN-300 shows exceptional stability over a broad pH range (1 to 14) in aqueous solutions, revealing the crucial contribution of the Cu-Pz coordination bond to its robustness. Significantly, PCN-300 demonstrates outstanding catalytic performance in the CDC reaction to form a C–O bond, achieving up to a 96% yield, arising from the synergistic effect of the 1D open channel, excellent stability, and the active Cu-porphyrin sites. Importantly, PCN-300 surpasses homogeneous Cu-porphyrin catalysts, showcasing superior catalytic performance, good recyclability, and high compatibility with various phenols, highlighting its potential as a regenerable heterogeneous catalyst. Control experiments confirm the synergistic interplay between the Cu-porphyrin center and framework in PCN-300 and computations unveil the free radical pathway of the catalytic reaction. Our work not only highlights the efficacy of stable Pz-based MOFs in catalyzing challenging organic transformations but also demonstrates the significance of synergistic effects between the MOF framework and active sites for efficient catalysis.

## Supporting Information Available

Experimental procedures, characterization methods, calculation methods, syntheses of PCN-300 and complex-TPPP, SEM and TEM images, X-ray diffraction results, X-ray photoelectron spectroscopy results, ^1^H NMR results, gas uptake analysis, thermogravimetric analysis, PCN-300 cif file, single-crystal data of PCN-300 (CCDC 2314509), complex-TPPP cif file, and single-crystal data of complex-TPPP (CCDC 2314510) (PDF)
