# Supplementary material for: A Robust Pyrazolate Metal–Organic Framework for Efficient Catalysis of Dehydrogenative C–O Cross Coupling Reaction
Source: J Am Chem Soc. 2024 May 9;146(20):14174–81. doi: 10.1021/jacs.4c03038 (PMC11117398; doi:10.1021/jacs.4c03038)
Supplement: Supplementary file 1 — ja4c03038_si_001.pdf [file ja4c03038_si_001.pdf]

Supporting Information

A robust pyrazolate metal-organic framework for efficient catalysis of dehydrogenative C–O cross coupling reaction

Rong-Ran Liang,[#] Zongsu Han,[#] Peiyu Cai, Yihao Yang, Joshua Rushlow, Zhaoyi Liu, Kun-Yu Wang*, and Hong-Cai Zhou*

Department of Chemistry, Texas A&M University, College Station, TX 77843, United States.

Contents

Section 1. General methods and instruments 4

Section 2. MOF Synthesis and Catalysis Experiment 5

Section 3. MOF Synthesis and Characterizations of the MOFs 5

Figure S1. The inclined angle and distance between adjacent layers in PCN-300 5

Figure S2. Packing structure of PCN-300 6

Figure S3. Demonstration of the solvent accessible void of PCN-300. 6

Figure S4. The connecting node between H_4TPPP and its extension in complex-TPPP 7

Figure S5. Packing structure of complex-TPPP 7

Figure S6. Demonstration of the solvent accessible void of complex-TPPP. 8

Figure S7. TEM images of PCN-300 and complex-TPPP 8

Figure S8. XPS spectra of PCN-300 9

Figure S9. XPS spectra of complex-TPPP. 9

Figure S10. CO_2 sorption isotherm, BET and Langmuir surface area plots of PCN-300 9

Figure S11. Nitrogen sorption isotherm, BET surface area plot, Langmuir surface area plot, and pore size distribution profile of complex-TPPP 10

Figure S12. TGA profile and PXRD patterns of PCN-300 10

Figure S13. PXRD patterns of complex-TPPP 10

Figure S14. The reaction conversion and yield versus the DTBP equivalent. 10

Figure S15. ^{1}H NMR spectra of the samples from 1 + a reaction system after reacting for different time 11

Figure S16. ^{1}H NMR spectra of the samples from 1 + a reaction system with different ratio of PCN-300 11

Figure S17. ^{1}H NMR spectra of the samples from 1 + a reaction system with different DTBP equivalent 12

Figure S18. ^{1}H NMR spectra of the samples from 1 + a reaction system with different catalysts 12

Figure S19. PXRD patterns of PCN-602 and PCN-602-Cu 13

Figure S20. PXRD pattern of PCN-300-Ni 13

Figure S21. SEM image and TGA profile of PCN-300-Ni 13

Figure S22. Nitrogen sorption isotherm, BET surface area plot, and Langmuir surface area plot of PCN-300-Ni 13

Figure S23. XPS spectra of PCN-300-Ni 14

Figure S24. ^{1}H NMR spectra of the sample from 1 + a reaction system before and after separating PCN-300 catalyst 14

Figure S25. The reaction conversion and yield before and after separating PCN-300 catalyst 15

Figure S26. ^{1}H NMR spectra of the samples from 1 + a reaction system after different cycles 15

Figure S27. ^{1}H NMR spectra of substrate 1 and the sample from 1 + a reaction system 16

Figure S28. ^{1}H NMR spectra of substrate 2 and the sample from 2 + a reaction system 16

Figure S29. ^{1}H NMR spectra of substrate 3 and the sample from 3 + a reaction system 17

Figure S30. ^{1}H NMR spectra of substrate 4 and the sample from 4 + a reaction system............17
Figure S31. ^{1}H NMR spectra of substrate 5 and the sample from 5 + a reaction system............18
Figure S32. ^{1}H NMR spectra of substrate 6 and the sample from 6 + a reaction system............18
Figure S33. SEM images of PCN-300 and complex-TPPP 19
Figure S34. ^{1}H NMR spectra of substrate 1 and the samples from 1 + a reaction system with/without presence of BHT..19
Figure S35. Gibbs free energy profile for the CDC reaction 20
Table S1. Crystallographic data and structural refinement summary 20
Table S2. Summary of the reported catalysts for the CDC reaction of substituted phenols and *p*-dioxane 21
Reference..21

Section 1. General methods and instruments

Nuclear Magnetic Resonance (NMR) spectroscopy. ^{1}H NMR, and ^{13}C NMR spectra were obtained on a AVANCE NEO 400 spectrometer.

Powder X-ray diffraction (PXRD). PXRD patterns were collected on a Bruker D8 Advance ECO powder diffractometer with a Cu microfocus tube (λ = 1.54178 Å) at 40 kV and 25 mA.

Thermogravimetric analysis (TGA)
TGA measurement was conducted on a Mettler-Toledo TGA/DSC 1 under nitrogen (N_2) atmosphere with a ramp rate of 5 °C/min from room temperature and 800 °C.

Scanning electron microscopy (SEM)
SEM was carried out using a FEI QUANTA 600 FE-SEM scanning electron microscope. The samples were dispersed over the slices of silicon wafer adhered to flat copper platform sample holders and then coated with gold using a sputter coater (ambient temperature, 85 torr pressure in a nitrogen atmosphere, puttered for 30s from a solid gold target at a current at 30 mA) before being submitted to SEM characterization.

Transmission electron microscopy (TEM)
TEM and HR-TEM images were recorded on Titan Themis 300 S/TEM at an accelerating voltage of 300 kV.

X-ray photoelectron spectroscopy (XPS)
XPS measurements were conducted on the Omicron XPS/UPS system with Argus detector using Omicron's DAR 400 dual Mg/Al X-ray source.

Nitrogen (N_2) sorption measurement. N_2 adsorption-desorption measurement was performed on a Micromeritics ASAP 2020 system. Prior to the measurement, the as-synthesized samples were washed with *N*,*N*-dimethylformamide (DMF) to remove the unreacted starting materials, followed by the exchange with acetone for several times to remove the non-volatile DMF. The resulting samples were then activated under vacuum at 100 °C for 12 h. The N_2 adsorption-desorption isotherms were then measured at 77 K, from which the specific surface areas were generated using the Brunauer-Emmett-Teller (BET) and Langmuir models.

Single-crystal X-ray Crystallography.
The single crystals of PCN-300 and complex-TPPP were directly transferred from the mother liquid to the oil, and then mounted onto a loop for single crystal X-ray diffraction (SCXRD) measurements. The data were collected on a Bruker D8-Venture diffractometers equipped with Cu microfocus tubes (λ = 1.54178 Å) and low temperature device. The single crystal structures were solved and refined using Olex2 software.[1] Both structures were solved by the direct method using the *SHELXT* program and refined by full-matrix least-squares method with *SHELXL* package.[2] All non-hydrogen atoms were refined with anisotropic displacement parameters, and the hydrogen atoms were positioned by geometrical calculation and then refined by riding. The free solvent molecules are highly disordered in MOFs and attempts to locate and refine the solvent peaks were unsuccessful. The diffused electron densities resulting from these solvent molecules were removed using the solvent MASK. Crystal data are summarized in Table S1 and the single crystal structures can be obtained free of charge from The Cambridge Crystallographic Data Centre with the CCDC number of 2314509 (PCN-300) and 2314510 (Complex-TPPP).

Calculation methods
All density functional theory (DFT) calculations were performed using ORCA software package (version 5.0.4).[3] The geometry optimization for all molecules was carried out at r^{2}SCAN-3c/def2-mTZVVP/def2-mTZVVP/J[4] level of theory. All calculations were corrected utilizing the geometrical counterpoise correction gCP[5] and the atom-pairwise dispersion correction based on tight binding partial charges (D4).[6] Frequency calculations were performed to validate each structure at minimum (no imaginary frequencies). The free energies for all structures were calculated at 298.15 K and 1.0 atm using the same level as geometry optimization.

Section 2. MOF Synthesis and Catalysis Experiment

Synthesis of PCN-300.

H_4TPPP (10 mg), $Cu(NO_3)_2{\cdot}6H_2O$ (40 mg), hydrochloric acid (500 μL), *N*,*N*-dimethylformamide (DMF) (5 mL) and methanol (2 mL) were charged in a 20 mL Pyrex vial. After sonication of around 10 minutes, the mixture was then heated at 80 °C for 1 day to give PCN-300 (yield: ~60 %).

Synthesis of complex-TPPP.

H_4TPPP (5 mg), $Cu(OAc)_2$ (10 mg), acetic acid (400 μL), DMF (1 mL) and water (1 mL) were charged in a 4 mL Pyrex vial. After sonication of around 10 minutes, the mixture was then heated at 120 °C for 3 days to give complex-TPPP (yield: ~50 %).

Synthesis of PCN-602-Cu.

H_4TPPP (10 mg), $CuCl_2$ (20 mg), and DMF (2 mL) were charged in a 4 mL Pyrex vial and heated at 80 °C for 1 day. After filtration and washed with methanol, the powder was dried to give H_4TPPP-Cu. H_4TPPP-Cu (10 mg), $Ni(OAc)_2{\cdot}4H_2O$ (10 mg), DMF (2 mL) and water (1.2 mL) were charged in a 4 mL Pyrex vial. After sonication of around 10 minutes, the mixture was then heated at 120 °C for 1 day to give PCN-602-Cu (yield: ~65 %).

Synthesis of PCN-300-Ni.

H_4TPPP (10 mg), $Ni(OAc)_2{\cdot}4H_2O$ (20 mg), and DMF (2 mL) were charged in a 4 mL Pyrex vial and heated at 80 °C for 1 day. After filtration and washed with methanol, the powder was dried to give H_4TPPP-Ni. H_4TPPP-Ni (10 mg), $Cu(NO_3)_2{\cdot}6H_2O$ (40 mg), hydrochloric acid (500 μL), *N*,*N*-dimethylformamide (DMF) (5 mL) and methanol (2 mL) were charged in a 20 mL Pyrex vial. After sonication of around 10 minutes, the mixture was then heated at 80 °C for 1 day to give PCN-300 (yield: ~62 %).

Catalysis experiment.

Prior to the catalysis experiments, the as-synthesized PCN-300 sample was washed with acetone several times, followed by vacuuming at 80 °C overnight. The catalysis experiments were performed using the following general procedures. A solution of substituted phenol substrate (**1**-**6**, 0.25 mmol) in 1,4-dioxane (2 mL) was added various catalysts and di-*tert*-butyl peroxide (DTBP) in high-pressure flask. After heating at 120 °C for a given time, the reaction mixture was cooled to room temperature and filtered, the filtrate was then concentrated in vacuum and monitored through NMR measurements.

Section 3. MOF Synthesis and Characterizations of the MOFs

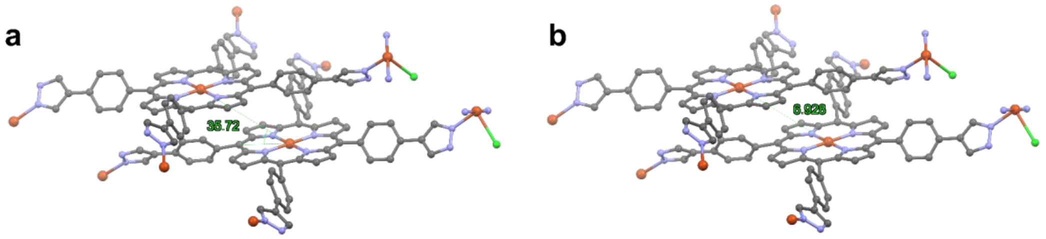

Figure S1. Demonstration of (a) the inclined angle and (b) the distance between adjacent layers in PCN-300.

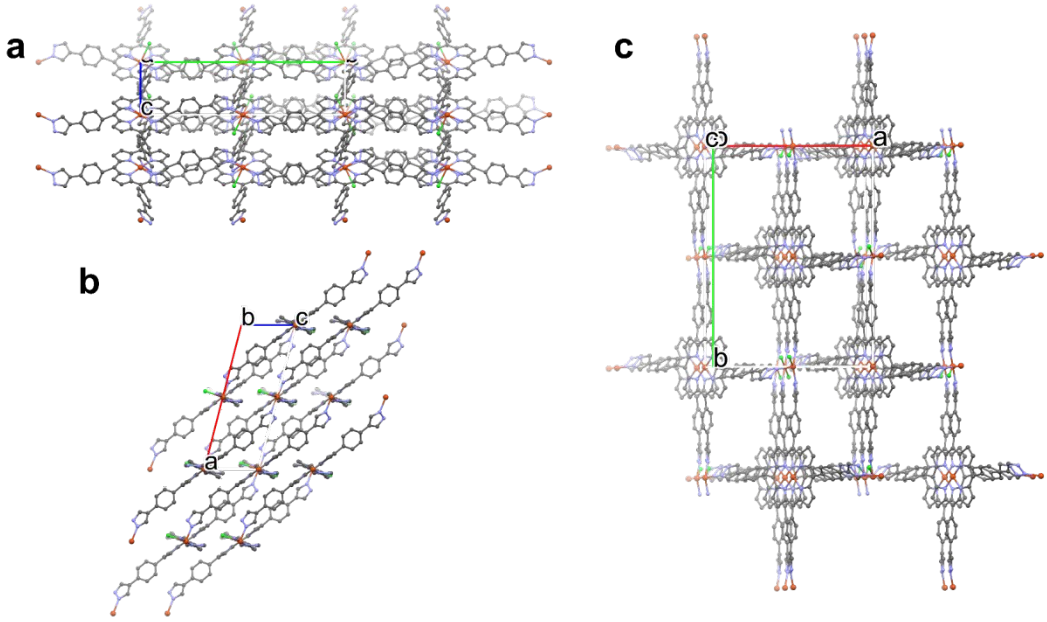

Figure S2. Packing structure of PCN-300 along (a) *a* axis, (b) *b* axis, and (c) *c* axis.

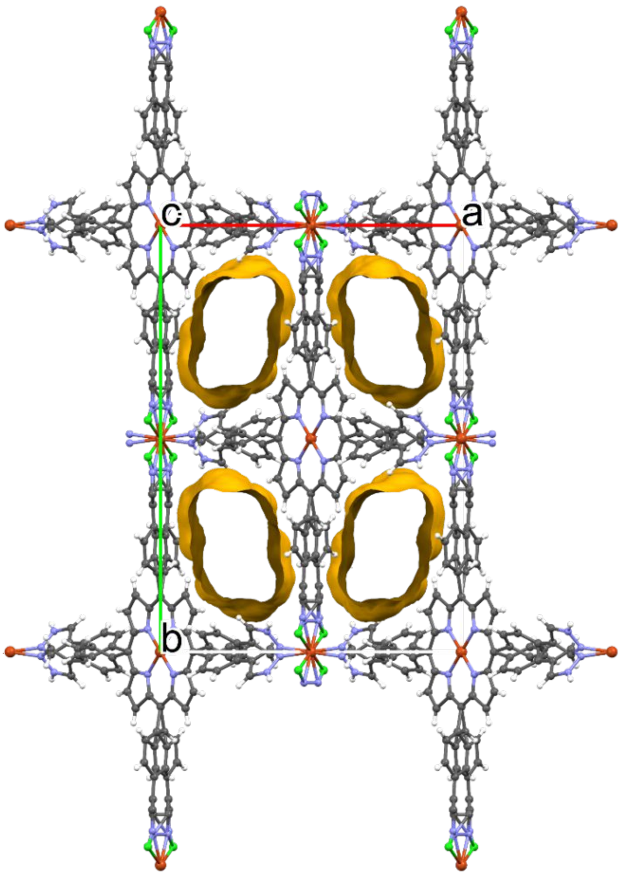

Figure S3. Demonstration of the solvent accessible void of PCN-300.

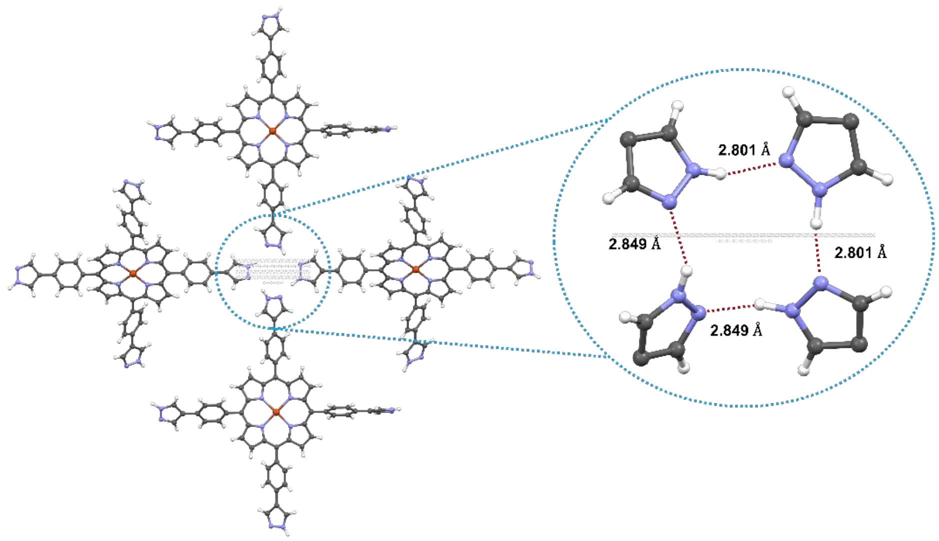

Figure S4. The connecting node between H_4TPPP and its non-planar extension in complex-TPPP.

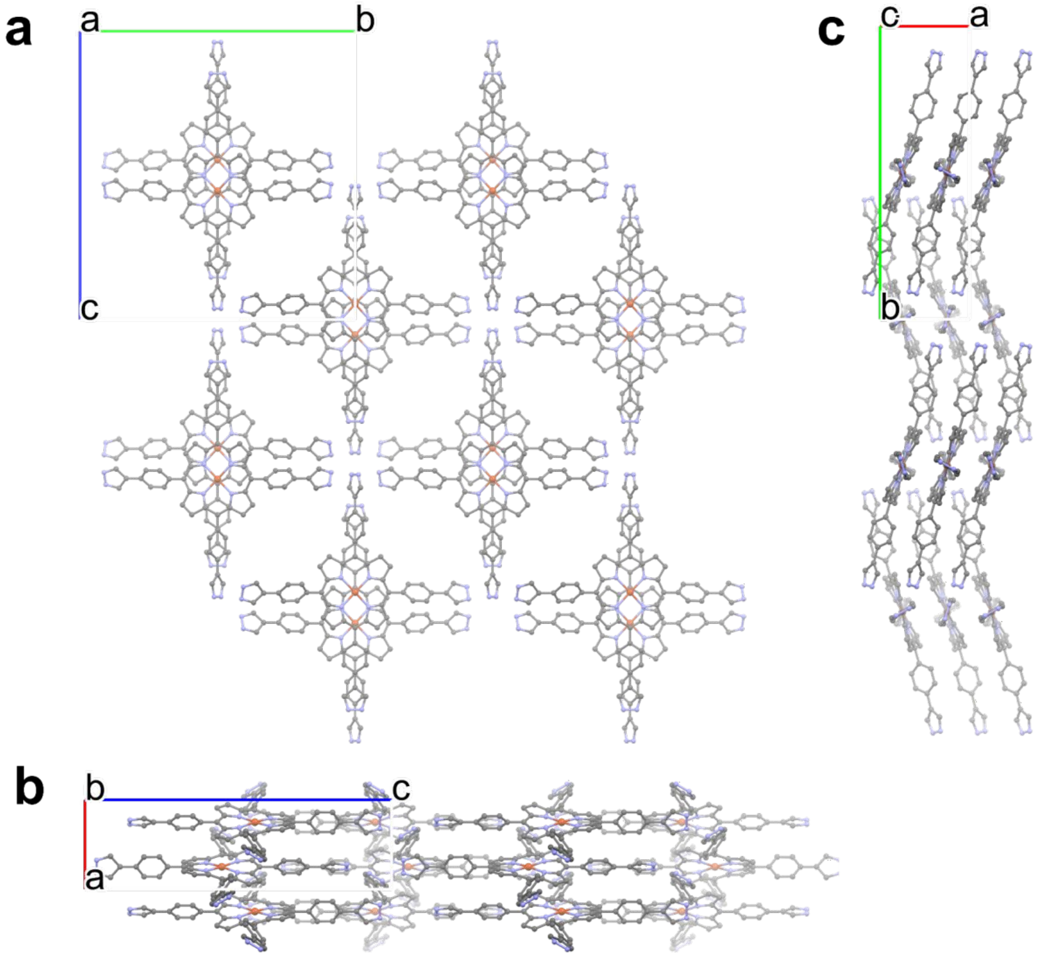

Figure S5. Packing structure of complex-TPPP along (a) *a* axis, (b) *b* axis, and (c) *c* axis.

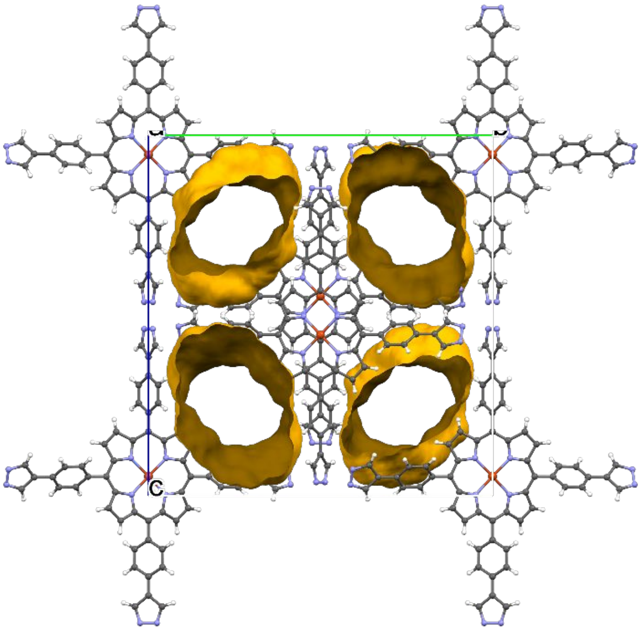

Figure S6. Demonstration of the solvent accessible void of complex-TPPP.

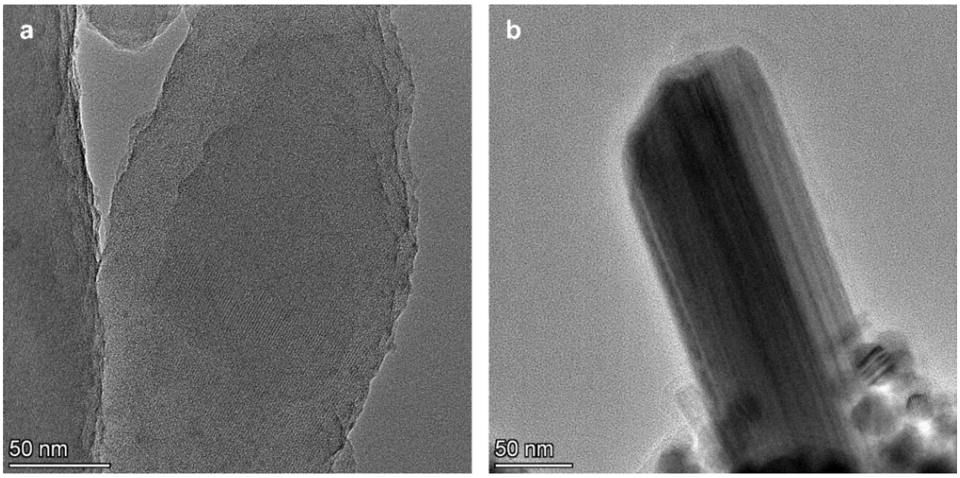

Figure S7. Transmission electron microscopy images of (a) PCN-300 and (b) complex-TPPP.

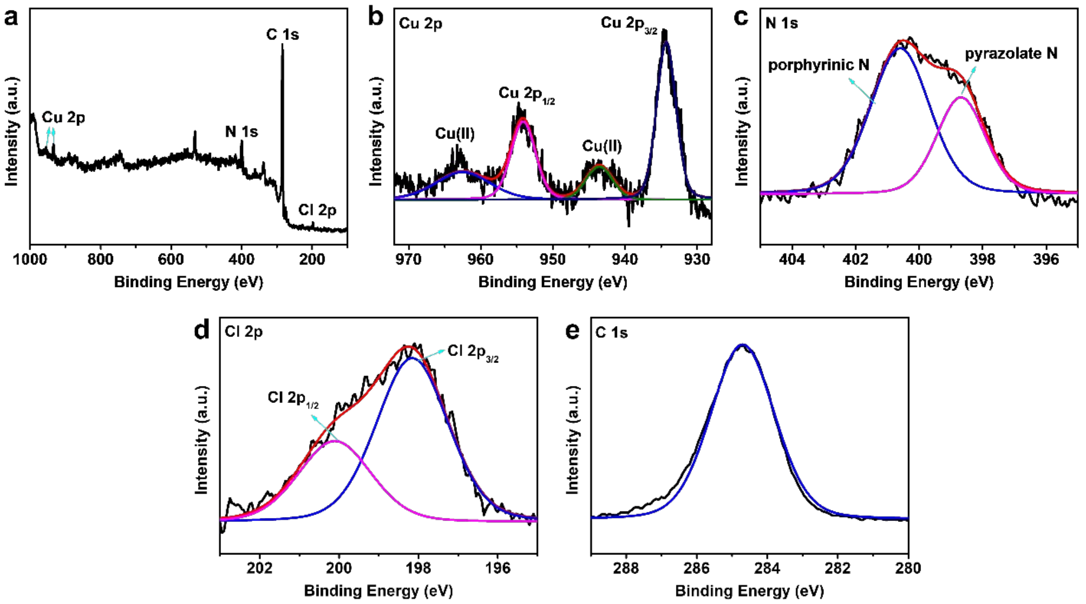

Figure S8. (a) XPS survey spectrum, and high-resolution XPS scan spectra over (b) Cu 2p, (c) N 1s, (d) Cl 2p, and (e) C 1s peaks of PCN-300.

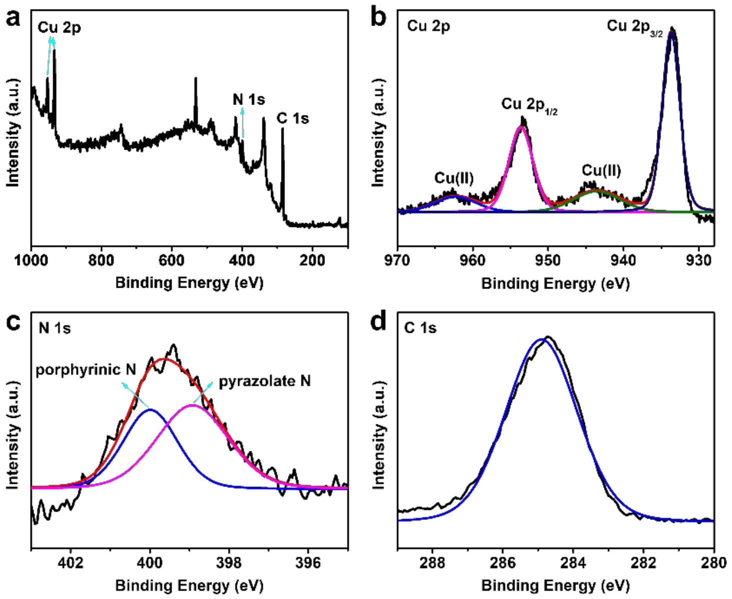

Figure S9. (a) XPS survey spectrum, and high-resolution XPS scan spectra over (b) Cu 2p, (c) N 1s, and (d) C 1s peaks of complex-TPPP.

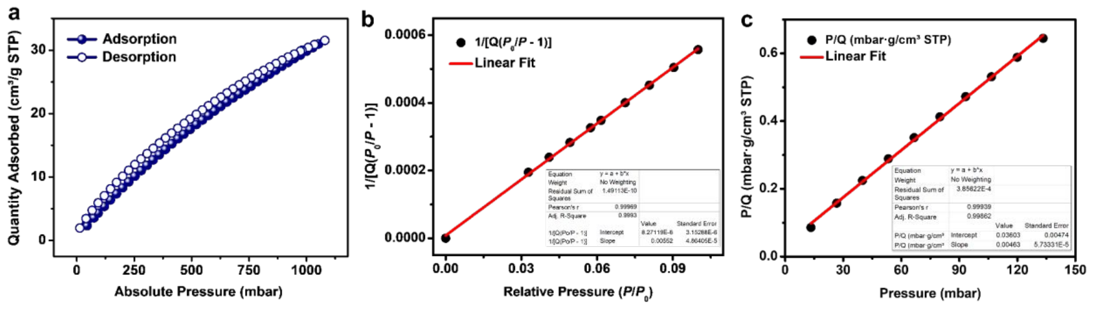

Figure S10. (a) CO_2 sorption isotherm at 298K, (b) BET surface area plot, and (c) Langmuir surface area plot of PCN-300.

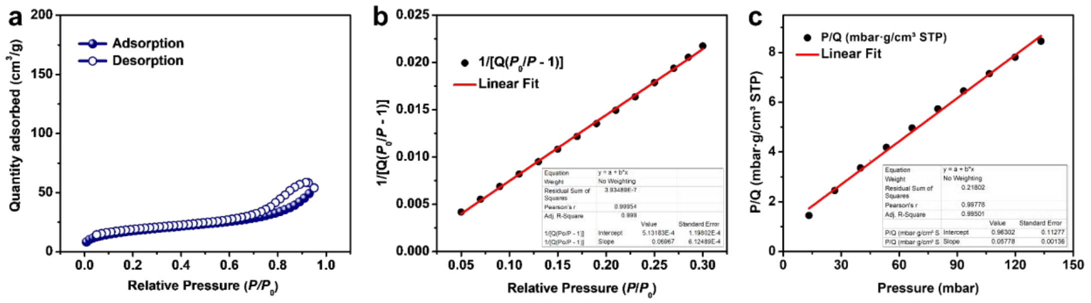

Figure S11. (a) Nitrogen sorption isotherm at 77K, (b) BET surface area plot, and (c) Langmuir surface area plot of complex-TPPP.

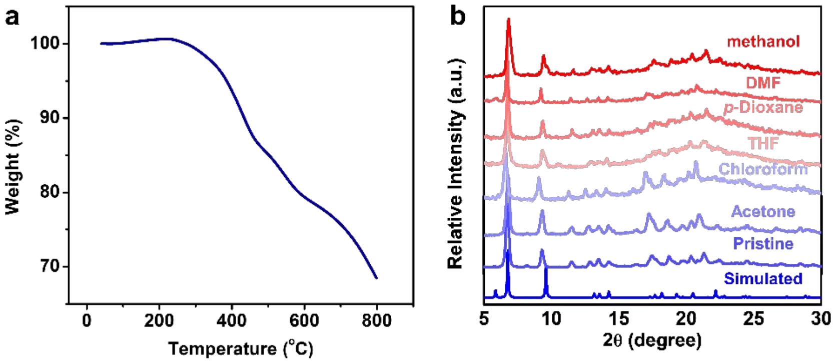

Figure S12. (a) TGA profile of PCN-300. (b) PXRD patterns of PCN-300 before and after immersing in various organic solvents.

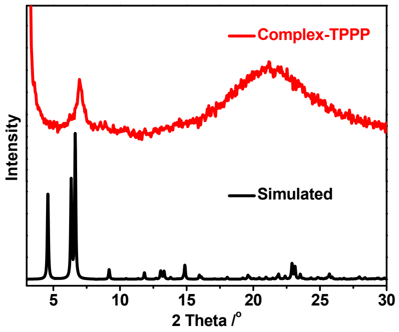

Figure S13. PXRD patterns of complex-TPPP.

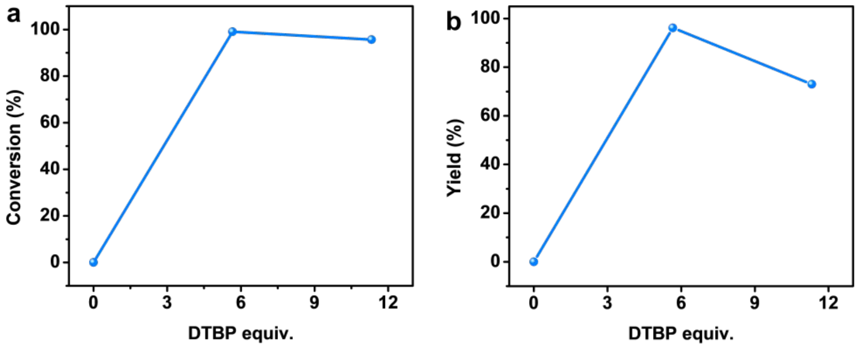

Figure S14. The reaction conversion (a) and yield (b) versus the DTBP equivalent. Note: Methyl 4-hydroxybenzoate and *p*-dioxane were chosen as substrates in the model reaction.

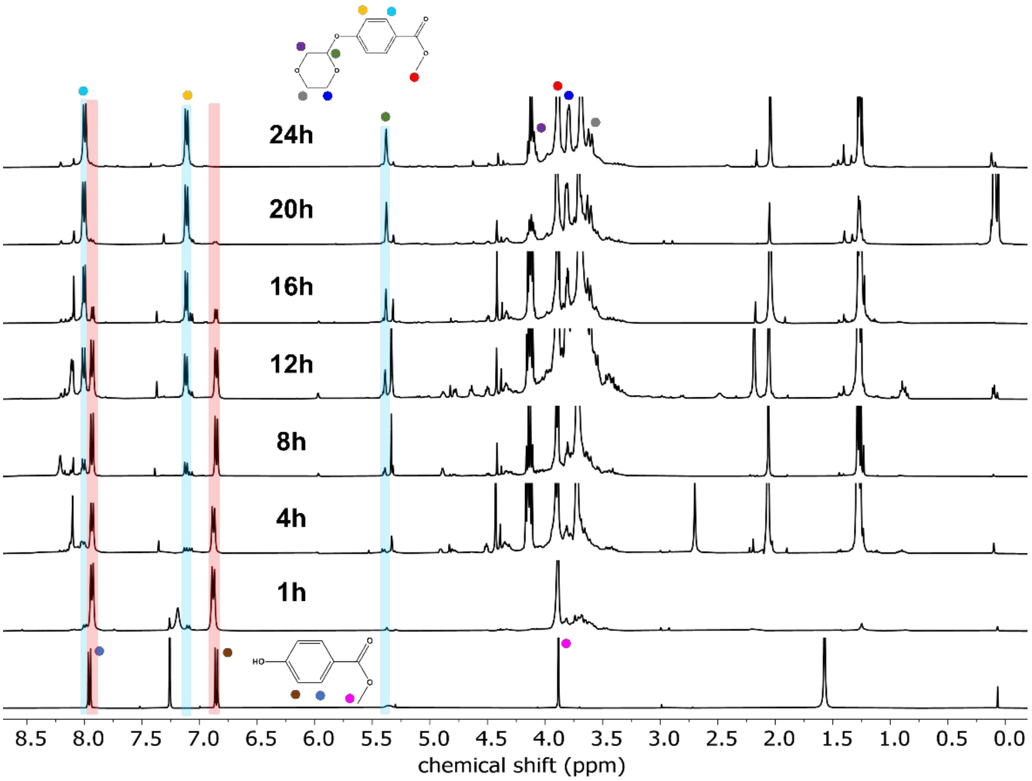

Figure S15. ^{1}H NMR spectra of the samples from **1** + **a** reaction system after reacting for different time.

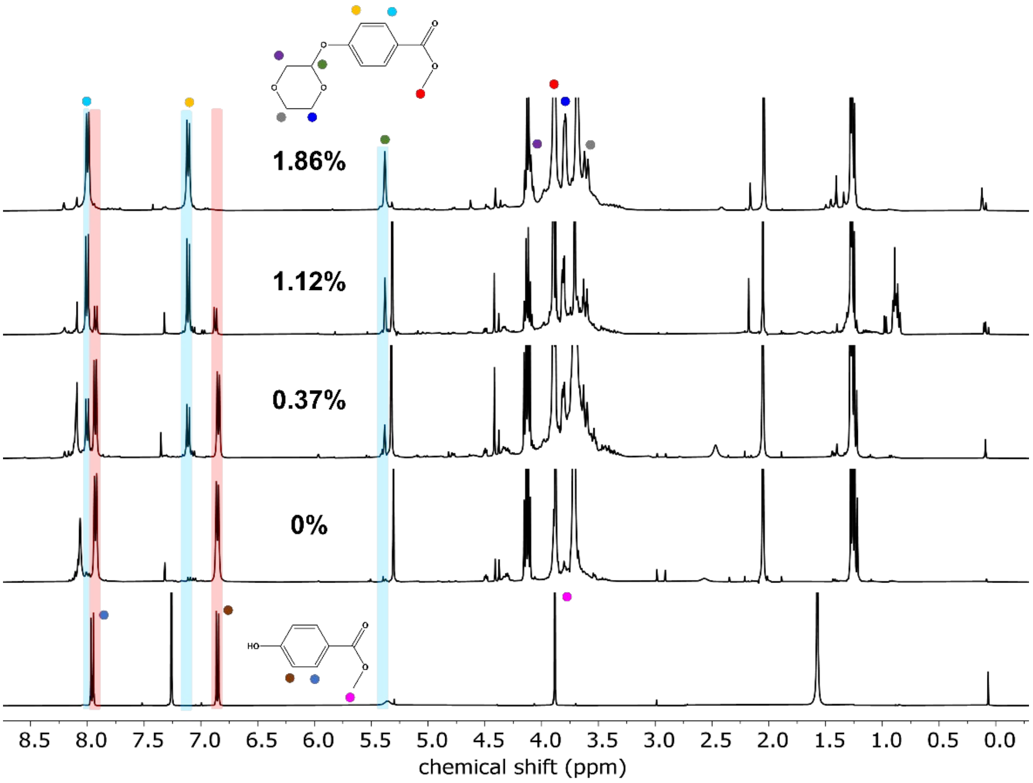

Figure S16. ^{1}H NMR spectra of the samples from **1** + **a** reaction system with different ratio of PCN-300.

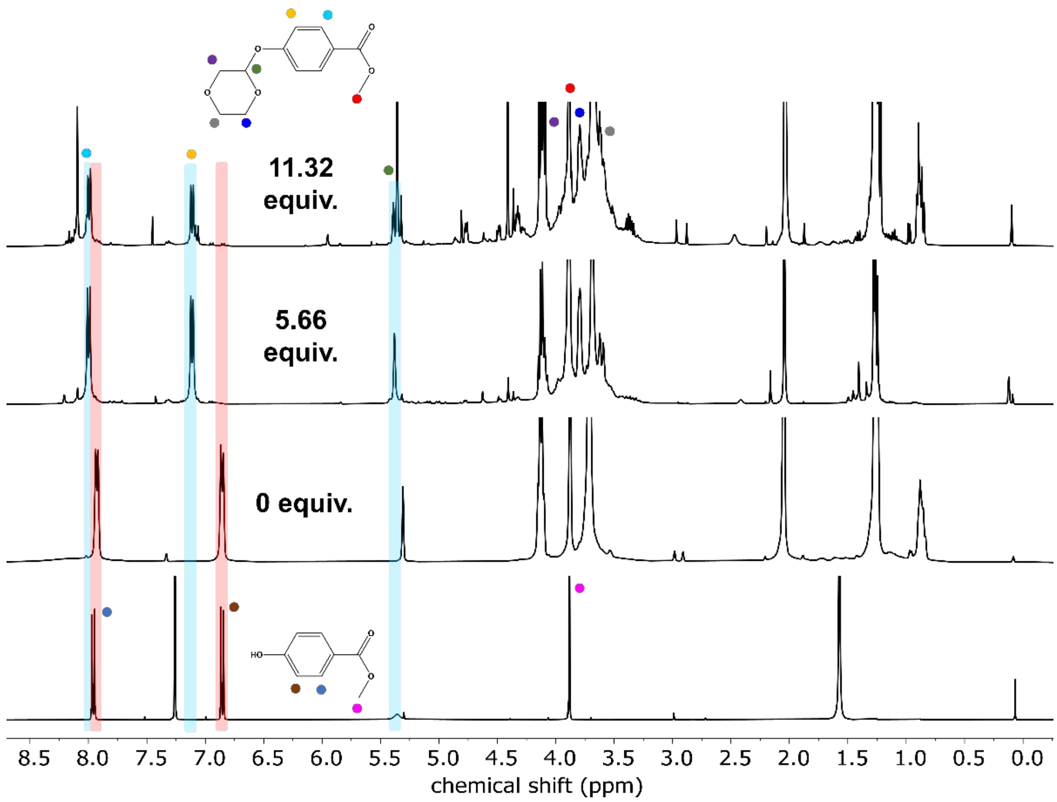

Figure S17. ^{1}H NMR spectra of the samples from **1** + **a** reaction system with different DTBP equivalent.

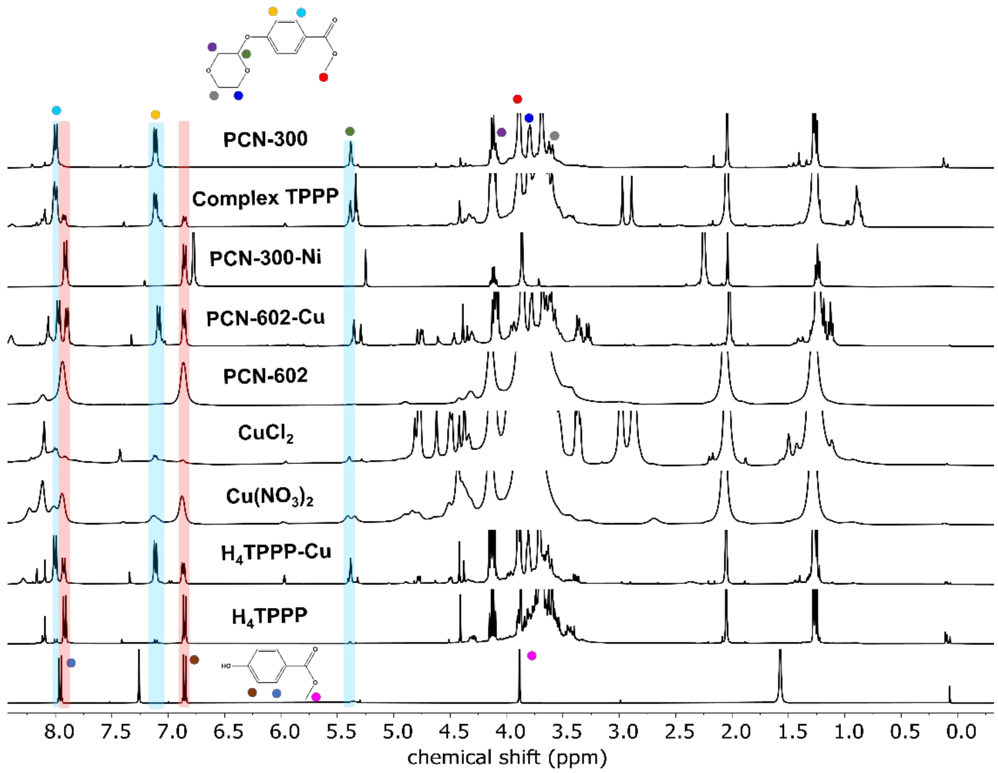

Figure S18. ^{1}H NMR spectra of the samples from **1** + **a** reaction system with different catalysts.

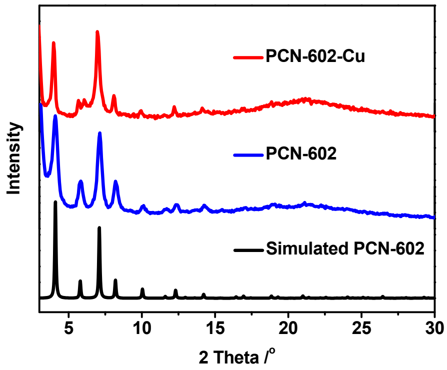

Figure S19. PXRD patterns of PCN-602 and PCN-602-Cu.

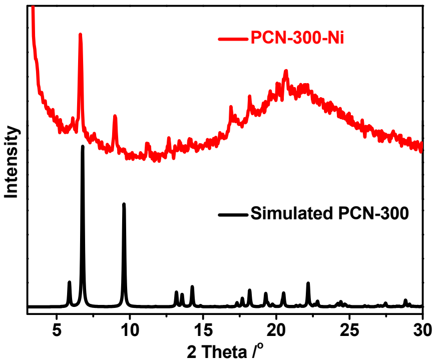

Figure S20. PXRD pattern of PCN-300-Ni.

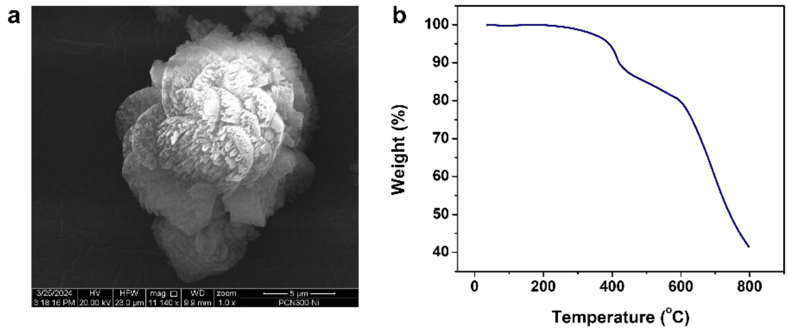

Figure S21. (a) SEM image and (b) TGA profile of PCN-300-Ni.

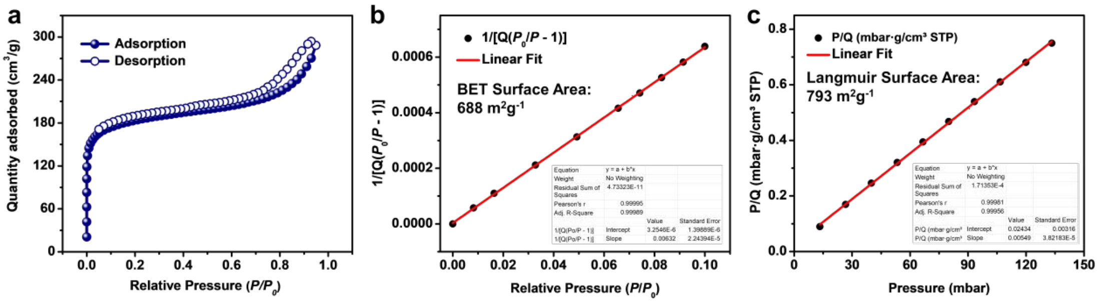

Figure S22. (a) Nitrogen sorption isotherm, (b) BET surface area plot, and (c) Langmuir surface area plot of PCN-300-Ni.

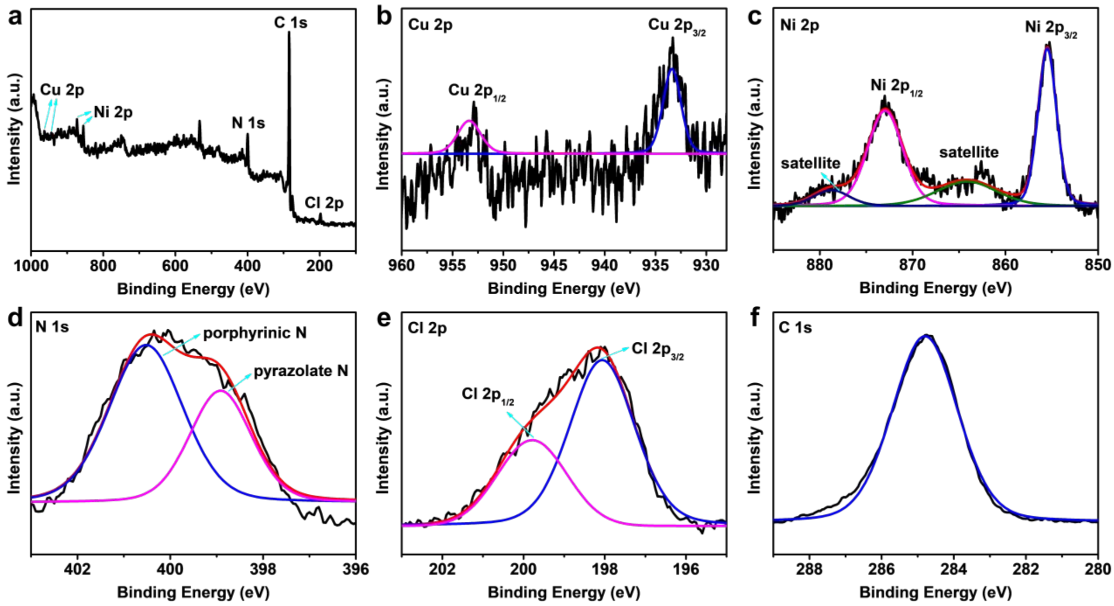

Figure S23. (a) XPS survey spectrum, and high-resolution XPS scan spectra over (b) Cu 2p, (c) Ni 2p, (d) N 1s, (e) Cl 2p, and (f) C 1s peaks of PCN-300-Ni.

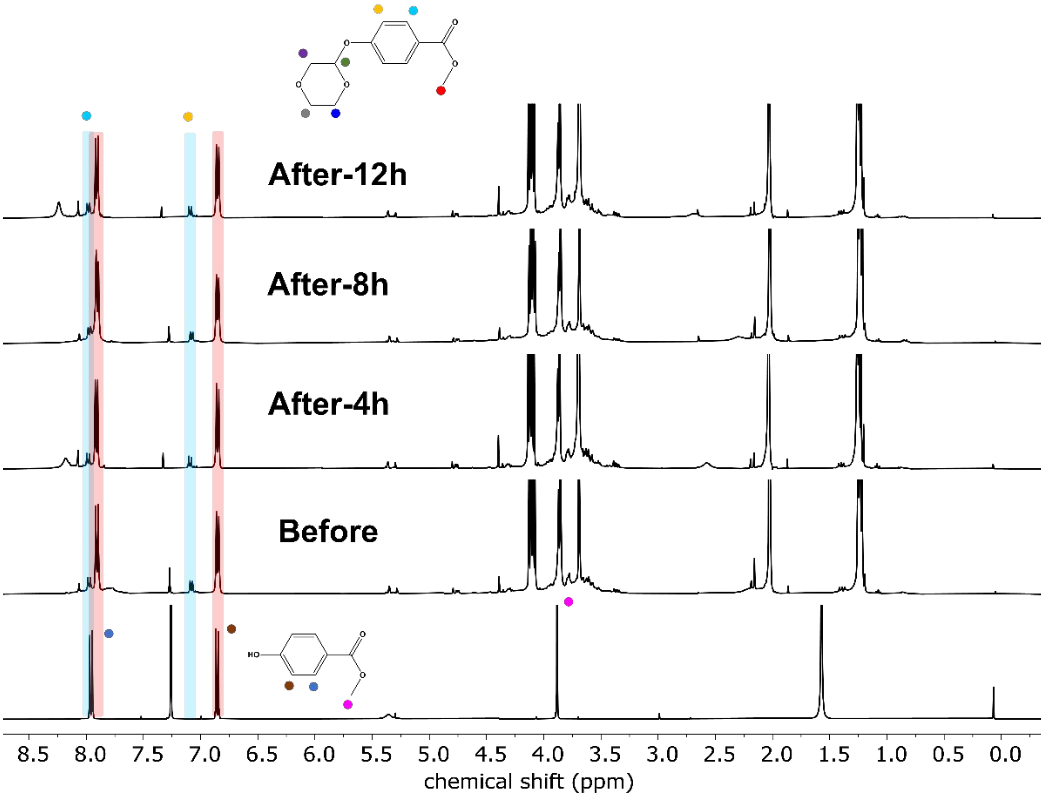

Figure S24. ^{1}H NMR spectra of the sample from **1** + **a** reaction system before and after separating PCN-300 catalyst. Note: The reaction was conducted under the optimal reaction condition of 1 (0.25 mmol), a (2 mL), PCN-300 (1.86 mol%), and DTBP (5.66 equiv.) at 120 ^oC.

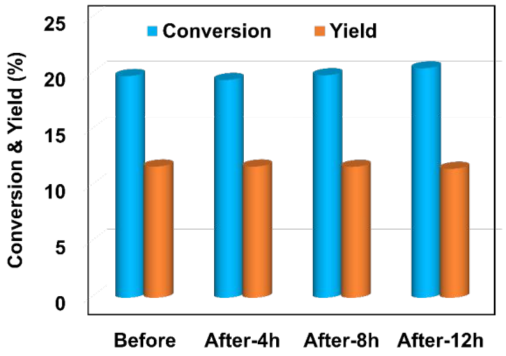

Figure S25. The reaction conversion and yield before and after separating PCN-300 catalyst. Note: The reaction was conducted under the optimal reaction condition of 1 (0.25 mmol), a (2 mL), PCN-300 (1.86 mol%), and DTBP (5.66 equiv.) at 120 ^{o}C.

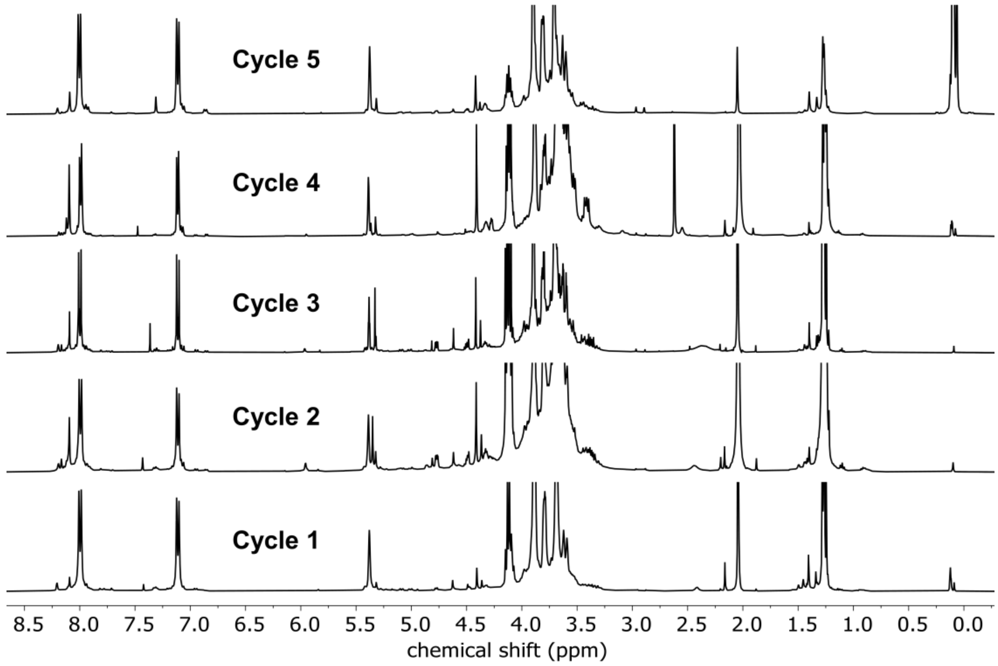

Figure S26. ^{1}H NMR spectra of the samples from **1** + **a** reaction system after different cycles.

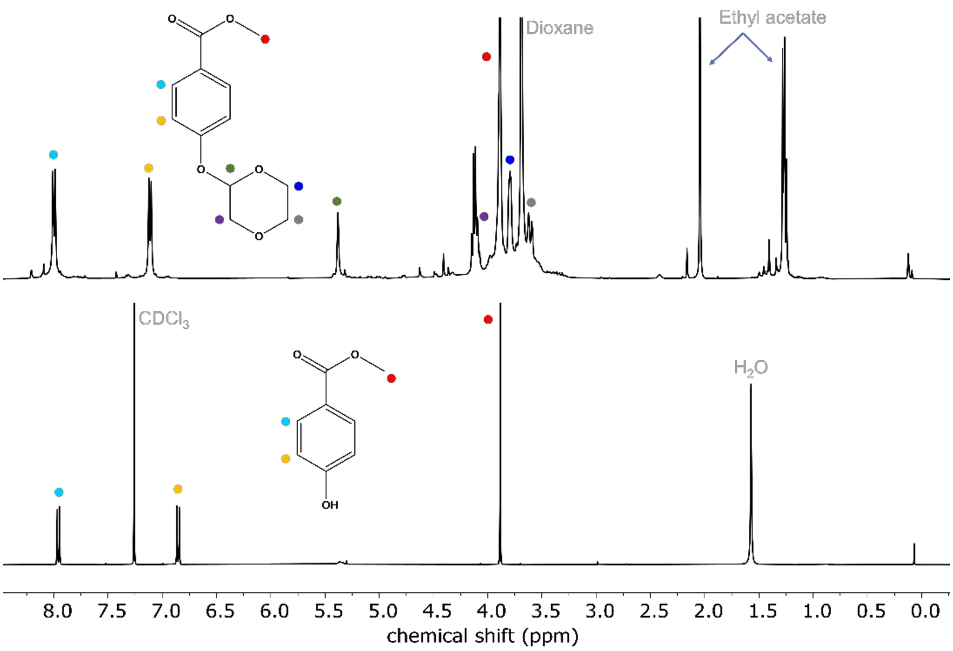

Figure S27. ^{1}H NMR spectra of substrate **1** and the sample from **1** + **a** reaction system.

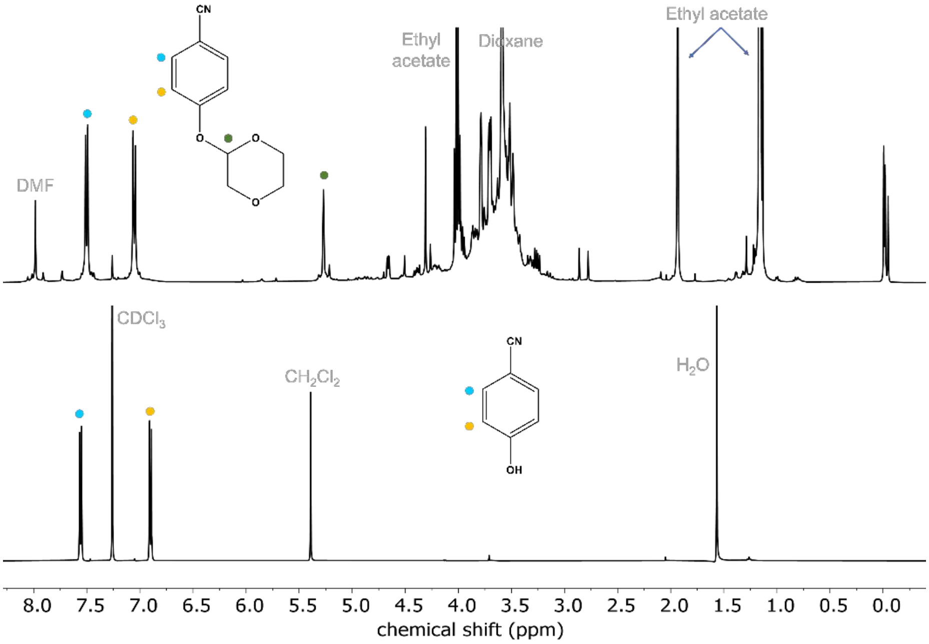

Figure S28. ^{1}H NMR spectra of substrate **2** and the sample from **2** + **a** reaction system.

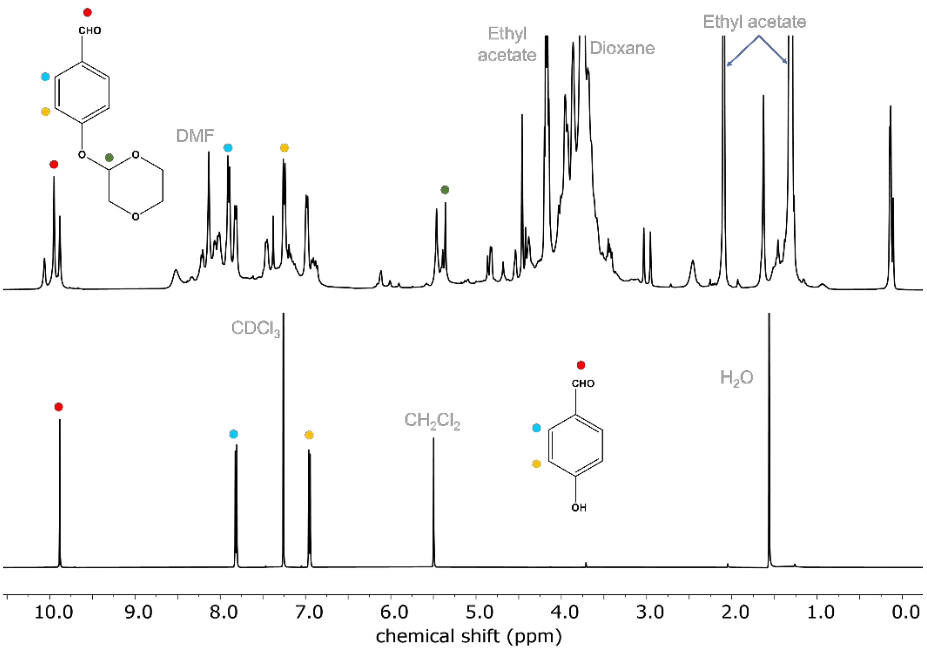

Figure S29. ^{1}H NMR spectra of substrate **3** and the sample from **3** + **a** reaction system.

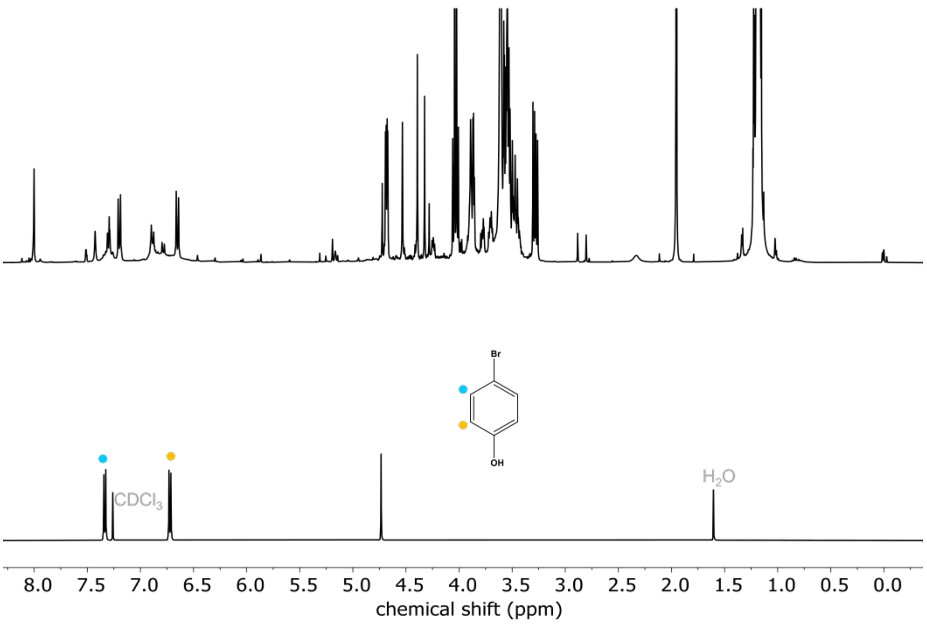

Figure S30. ^{1}H NMR spectra of substrate **4** and the sample from **4** + **a** reaction system.

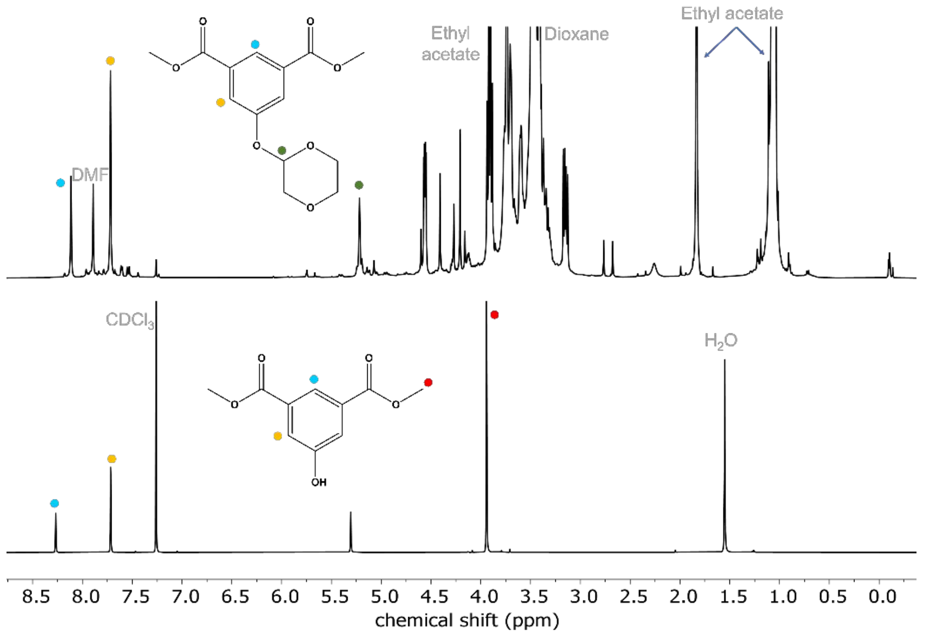

Figure S31. ^{1}H NMR spectra of substrate **5** and the sample from **5** + **a** reaction system.

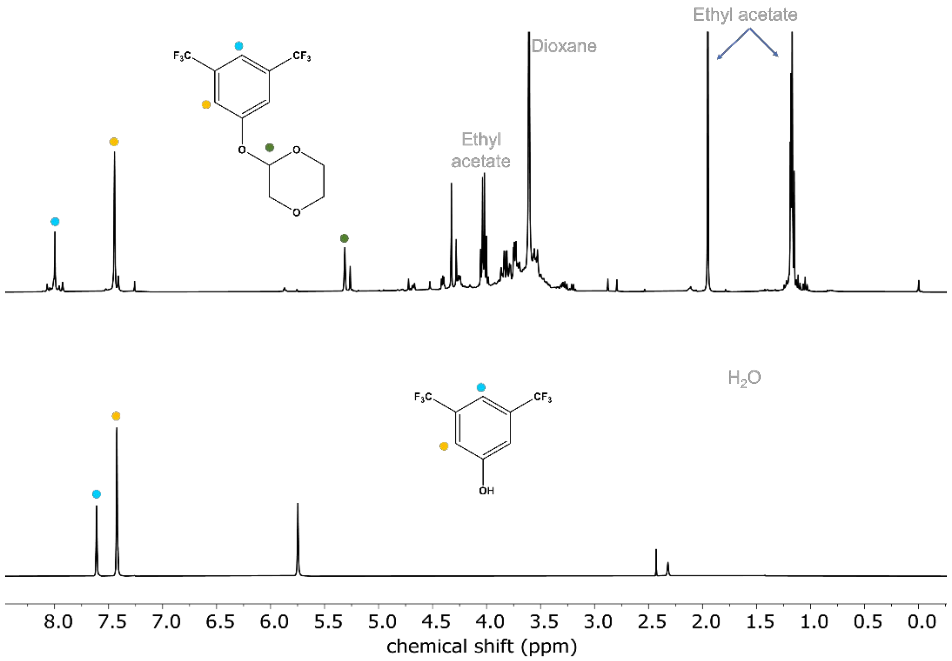

Figure S32. ^{1}H NMR spectra of substrate **6** and the sample from **6** + **a** reaction system.

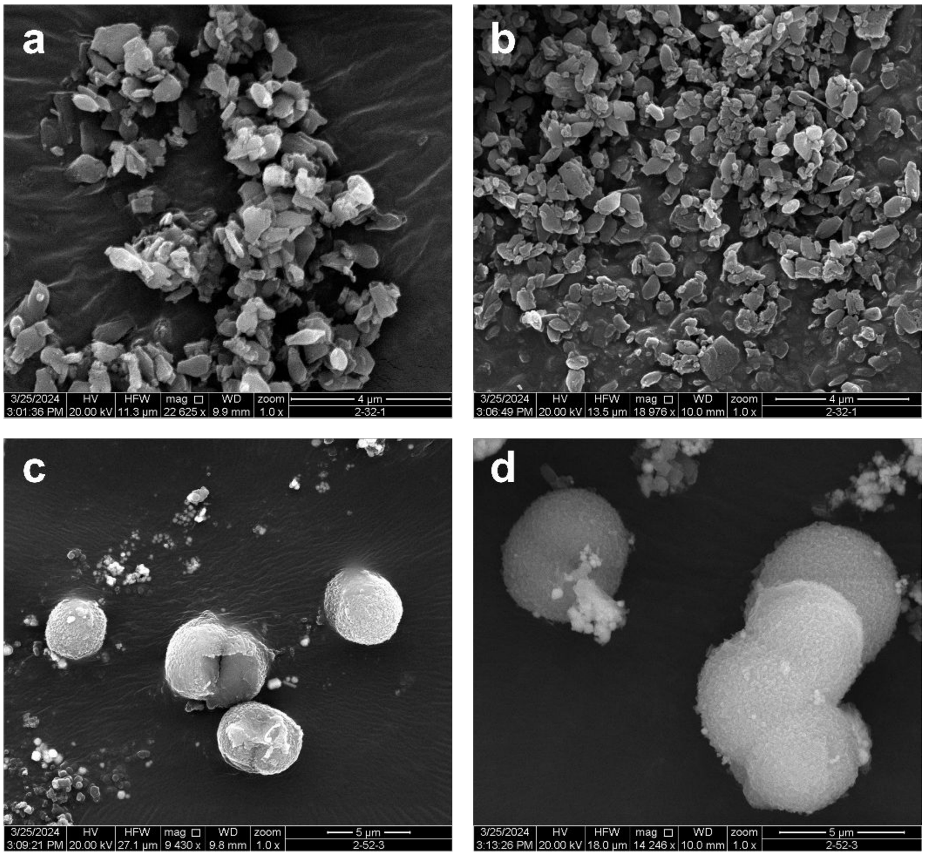

Figure S33. SEM images of PCN-300 before (a) and after (b) the catalytic cycles, and complex-TPPP before (c) and after (d) catalysis.

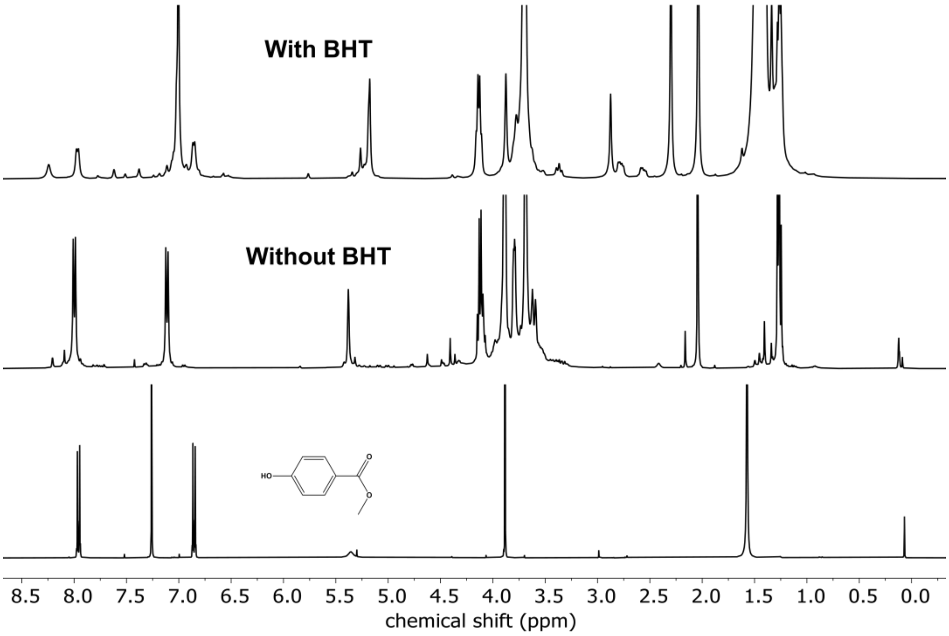

Figure S34. ^{1}H NMR spectra of substrate **1** and the samples from **1** + **a** reaction system with/without the presence of BHT.

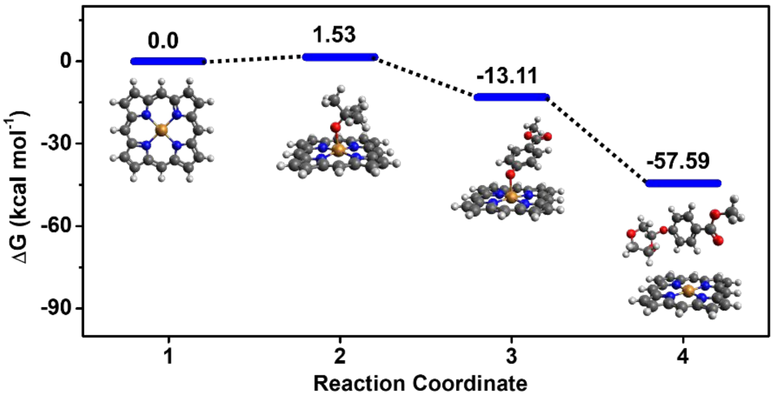

Figure S35. Gibbs free energy profile for the CDC reaction. The free energy of each intermediate state is relative to the free energy (zero) of the Cu-porphyrin in PCN-300.

Table S1. Crystallographic data and structural refinement summary.

Identification code	PCN-300	Complex-TPPP
CCDC	2314509	2314510
Empirical formula	$C_{28}H_{15}ClCuN_6$	$C_{56}H_{36}CuN_{12}$
Formula weight	534.45	940.51
Temperature/K	100	100.01(11)
Crystal system	monoclinic	orthorhombic
Space group	*C*2/*m*	*Pnna*
a/Å	18.9931(18)	8.1112(3)
b/Å	26.081(3)	26.5911(8)
c/Å	6.9278(7)	27.8600(10)
α/°	90	90
β/°	104.377(7)	90
γ/°	90	90
Volume/Å^3	3324.2(6)	6009.0(4)
Z	4	4
ρ_{calc} g/cm^3	1.068	1.040
μ/mm^{-1}	0.806	0.839
F(000)	1084.0	1940.0
Crystal size/mm^3	0.1 × 0.1 × 0.1	0.1 × 0.1 × 0.1
Radiation	synchrotron (λ = 0.7288)	Cu Kα (λ = 1.54184)
2θ range for data collection/°	3.202 to 43.122	13.812 to 109.118
Index ranges	$-19 \leq h \leq 19, -26 \leq k \leq 26, -6 \leq l \leq 6$	$-8 \leq h \leq 8, -28 \leq k \leq 27, -22 \leq l \leq 29$
Reflections collected	15856	18504
Independent reflections	1821 [R_{int} = 0.1906, R_{sigma} = 0.1075]	3658 [R_{int} = 0.0873, R_{sigma} = 0.0632]
Data/restraints/parameters	1821/252/248	3658/0/316
Goodness-of-fit on F^2	1.131	1.002
Final R indexes [I>=2σ (I)]	R_1 = 0.0938, wR_2 = 0.2686	R_1 = 0.0713, wR_2 = 0.2316
Final R indexes [all data]	R_1 = 0.1400, wR_2 = 0.3131	R_1 = 0.0907, wR_2 = 0.2526
Largest diff. peak/hole / e Å^{-3}	0.56/-0.67	0.63/-0.52

Table S2. Summary of the reported catalysts for the CDC reaction of substituted phenols and *p*-dioxane.

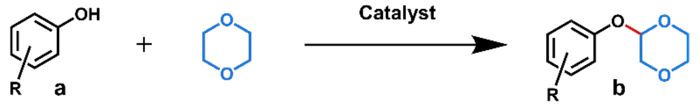

a	Catalyst (mol%)	Catalyst type	Oxidant (equiv.)	Yield (%)	Ref
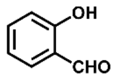	$Cu_2(BPDC)_2(BPY)$ (3)	Heterogeneous	*t*BuOOH (3)	100[a]	7
	1′ (1wt%)	Heterogeneous	*t*BuOOH (3)	96	8
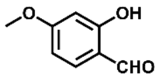	1′ (1wt%)	Heterogeneous	*t*BuOOH (3)	92	8
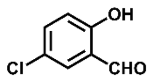	1′ (1wt%)	Heterogeneous	*t*BuOOH (3)	84	8
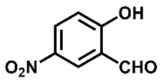	1′ (1wt%)	Heterogeneous	*t*BuOOH (3)	65	8
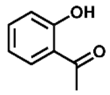	$[Cu(2\text{-pymo})_2]$) (5)	Heterogeneous	*t*BuOOH (1.5)	82	9
	($[Cu(im)_2]$ (5)	Heterogeneous	*t*BuOOH (1.5)	83	9
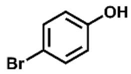	CuTECP (5)	Homogeneous	DTBP (3.5)	0	10
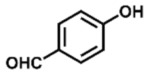	CuTECP (5)	Homogeneous	DTBP (3.5)	42	10
	CuTPyP (3)	Homogeneous	DTBP (6)	20	11
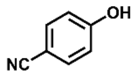	CuTECP (5)	Homogeneous	DTBP (3.5)	83	10
	CuTPyP (3)	Homogeneous	DTBP (6)	73	11
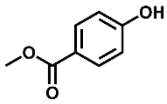	CuTECP (5)	Homogeneous	DTBP (3.5)	85	10
	CuTPP (3)	Homogeneous	DTBP (3.5)	33	11
	$CuF_{20}TPP$ (3)	Homogeneous	DTBP (3.5)	52	11
	CuT*n*PP (3)	Homogeneous	DTBP (3.5)	57	11
	CuT*t*BP (3)	Homogeneous	DTBP (3.5)	67	11
	CuTPyP (3)	Homogeneous	DTBP (6)	83	11

[a]Conversion

Reference

[1] O. V. Dolomanov, L. J. Bourhis, R. J. Gildea, J. A. K. Howard, H. Puschmann, *J. Appl. Cryst.* **2009**, *42*, 339-341.
[2] G. M. Sheldrick, *Acta Crystallogr. A* **2008**, *64*, 112-122.
[3] a) F. Neese, F. Wennmohs, U. Becker, C. Riplinger, *J. Chem. Phys.* **2020**, *152*, 224108; b) F. Neese, *WIREs Computational Molecular Science* **2022**, *12*, e1606.
[4] S. Grimme, A. Hansen, S. Ehlert, J.-M. Mewes, *J. Chem. Phys.* **2021**, *154*, 064103.
[5] H. Kruse, S. Grimme, *J. Chem. Phys.* **2012**, *136*, 154101.
[6] a) E. Caldeweyher, C. Bannwarth, S. Grimme, *J. Chem. Phys.* **2017**, *147*, 034112; b) E. Caldeweyher, S. Ehlert, A. Hansen, H. Neugebauer, S. Spicher, C. Bannwarth, S. Grimme, *J. Chem. Phys.* **2019**, *150*, 154122.
[7] N. T. S. Phan, P. H. L. Vu, T. T. Nguyen, *Journal of Catalysis* **2013**, *306*, 38-46.
[8] V. Sharma, D. De, P. K. Bharadwaj, *Inorg. Chem.* **2018**, *57*, 8195-8199.
[9] I. Luz, A. Corma, F. X. Llabrés i Xamena, *Catal. Sci. Technol.* **2014**, *4*, 1829-1836.
[10] S. Yang, M.-F. Xiong, W.-Q. Tian, H. Zhang, X.-Y. Xiao, H.-Y. Liu, C.-K. Chang, *Tetrahedron* **2020**, *76*, 131569.
[11] F.-H. Wang, Z.-Y. Liu, S. Yang, L. Shi, D.-Z. Lin, H.-Y. Liu, G.-Q. Yuan, *Synthetic Communications* **2021**, *51*, 2053-2062.
